# Supplementary material for: Simulation Study of the Effect of Antimicrobial Peptide Associations on the Mechanism of Action with Bacterial and Eukaryotic Membranes
Source: Membranes (Basel). 2022 Sep 16;12(9):891. doi: 10.3390/membranes12090891 (PMC9502835; doi:10.3390/membranes12090891)
Supplement: Supplementary file 1 [file membranes-12-00891-s001.zip › membranes-1846715-supplementary.pdf]

membranes

Supplementary materials

Simulation Study of the Effect of Antimicrobial Peptide Associations on the Mechanism of Action with Bacterial and Eukaryotic Membranes

Matko Maleš [1] and Larisa Zoranić [2,*]

[1] Faculty of Maritime Studies, University of Split, 21000 Split, Croatia; mmales@pfst.hr
[2] Department of Physics, Faculty of Science, University of Split, 21000 Split, Croatia
* Correspondence: larisaz@pmfst.hr

Abbreviations: AMP, antimicrobial peptide; MD, molecular dynamics; AA, all atom; CG, coarse-grained; POPE, 1-palmitoyl-2-oleoyl-sn-glycero-3-phosphoethanolamine; POPG, 1-palmitoyl-2-oleoyl-sn-glycero-3-phosphatidylglycerol; POPC, 1-palmitoyl-2-oleoyl-sn-glycero-3-phosphatidylcholine; Lys-PG, lysylphosphatidylglycerol; PVPE, 1-palmitoyl-2-cis-vaccenic-phosphatidylethanolamine; PVPG, 1-palmitoyl-2-cis-vaccenic-phosphatidylglycerol; PVCL2, 1,10-palmitoyl-2,20-vacenoyl-cardiolipin; LPS, lipopolysaccharide; 3D-HM, three-dimensional hydrophobic moment; COM, center of mass; OM, outer membrane.

List of figures and tables:

A) Models and molecular properties

Table S1 Initial simulation box sizes
Table S2 Properties of membrane systems
Figure S1 Adepantin-1 structure
Figure S2 Lipid and lipopolysaccharide models
Figure S3 2D-hydrophobic moment of adepantin-1

B) Peptides in AA-1 and AA-2 simulations

Figure S4 Snapshots of AA-1 Gram+ and Gram- simulations
Figure S5 Density profiles of AA-1 simulations
Figure S6 COM of polar and hydrophobic residues for AA-1 and AA-2 simulations
Table S3 3D-HM calculation in AA-1 simulations

C) Peptides in AA-12 simulations

Figure S7 Snapshots of AA-12 Gram- and Gram+ simulations
Figure S8 Cluster analysis and snapshots AA-12 Gram- simulations
Figure S9 Cluster analysis and snapshots AA-12 Gram+ simulations
Figure S10 Cluster analysis and snapshots AA-12 POPC simulations

D) Peptides in CG and CG2AA simulations

Figure S11 Initial configurations in CG simulations
Figure S12 Snapshots at early simulation time in CG simulations
Figure S13 Snapshots at the end simulation time in CG simulations
Figure S14 Representation of CG to AA procedure
Figure S15 Snapshots at the end simulation time in CG2AA simulations
Figure S16 Cluster analysis for 12 peptides, case CG-12 and CG2AA-12
Figure S17 Cluster analysis for 24 peptides, case CG-24 and CG2AA-24
Figure S18 Density profiles for AA-12 and CG2AA-12 cases

E) Membrane analysis

Figure S19.1-5 Area per lipid and membrane thickness time dependencies
Table S4 Details on area per lipid and membrane thickness
Figure S20.1-3 Area of lipid profiles for AA and CG simulations
Figure S21.1-2. Area of lipid and membrane thickness profiles for CG2AA simulations
Figure S22.1-3 Order parameters for all AA and CG2AA simulation experiments

F) Outer-membrane results

Figure S23 Structure of LPS molecule
Table S5 Details on area per lipid and membrane thickness
Figure S24 Area per lipid and membrane thickness time dependencies
Figure S25 Area of lipids profiles
Figure S26 Snapshots of OM 0 O-antigens cases
Figure S27 Snapshots of OM 2 O-antigens cases
Figure S28 Density profiles

G) DSSP profiles from AA and CG2AA simulation

Figure S29 Secondary structure in AA-1 simulations
Figure S30 Secondary structure in AA-2 simulations
Figure S31 Secondary structure in AA-12 Gram- simulations
Figure S32 Secondary structure in AA-12 Gram+ simulations
Figure S33 Secondary structure in AA-12 POPC simulations
Figure S34 Secondary structure in CG2AA simulations

A) Models and molecular properties

Table S1. Initial simulation box sizes.

Label	no. of peptides	Size of box (X × Y × Z) (nm)				
		Gram−	POPC	Gram+	Gram− outer membrane	
					2 O-Antigens	0 O-Antigens
AA-0	0					
AA-1a,b*	1	8.7 × 8.7 × 14.7	9.4 × 9.4 × 14.7	9.2 × 9.2 × 14.7	9.5 × 9.5 × 28.1	9.5 × 9.5 × 25.0
AA-2	2					
AA-12a,b*	12	12.4 × 12.4 × 14.7	13.2 × 13.2 × 16.0	13.0 × 13.0 × 14.7	13.0 × 13.0 × 23.1	13.0 × 13.0 × 26.1
CG-12	12	12.5 × 12.5 × 17.7				
CG-24	24					
CG2AA-12**	12	12.6 × 12.6 × 17.4				
CG2AA-24**	24					

* Case 1 simulations are marked with the letter a, and case 2 with the letter b; ** Last frame of CG simulations was transformed to atomistic model, and simulation continued as AA MD.

Table S2. Properties of membrane systems.

	APL* (nm^2)	APL (nm^2) (this work, 310 K)
POPE	0.58^{e} (308 K) [1]	0.58
POPG	0.66^{e} (303 K) [2]	0.65
POPC	0.64^{e} (303 K) [3]	0.64
Lys-PG	0.64^{s} (323 K) [4]	0.64
CL	1.15^{s} (313 K) [4]	0.77
POPE:POPG	0.61^{s} (310 K) [5]	0.59
PG:Lys-PG:CL	0.62^{s} (313 K) [4]	0.65

* e – experiment; s – MD simulations.

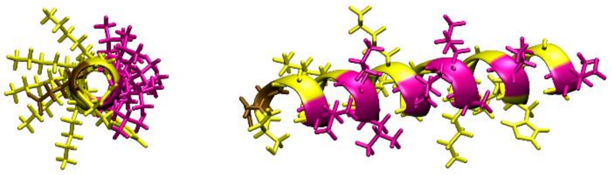

Figure S1. Initial structure for adepantin-1 (GIGKHVGKALKGLKGLLKGLGES–NH_2) as predicted by C-QUARK has strong amphipathic distribution of hydrophobic (magenta) and hydrophilic (yellow) residues [6]. C-QUARK prediction for adepantin-1 included 5 models: Model 1 with α-helical structure and highest score (estimated TM-score = 0.376 + -0.149) and other Models 2-5 with α-helical structure and a turn near 13th Leu amino acid.

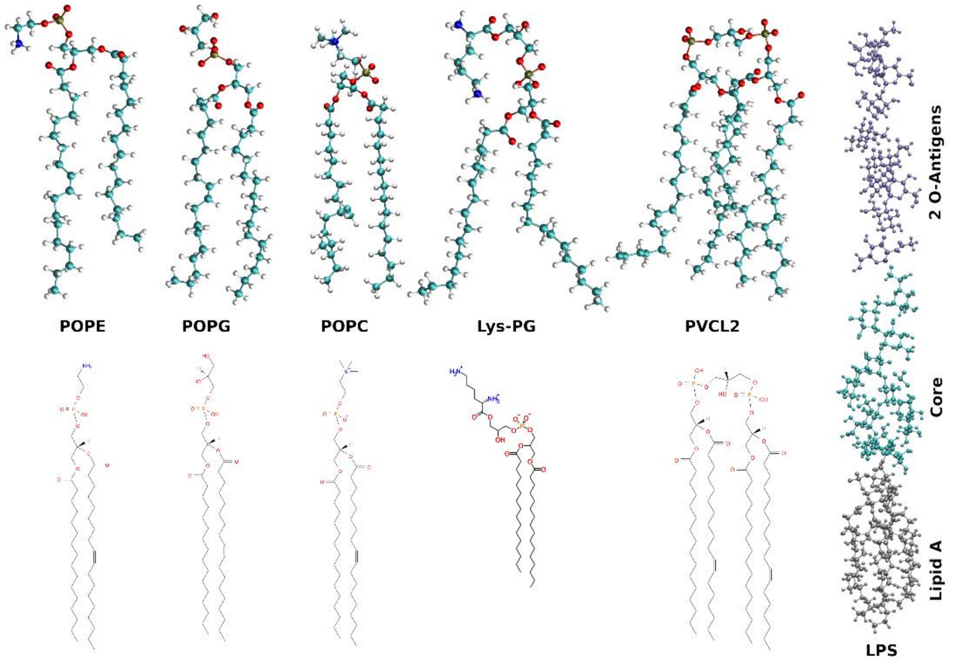

Figure S2. Lipid and lipopolysaccharide (LPS) molecules used in simulations from CHARMM-GUI database [7–9]. More details on the LPS are presented in Figure S23.

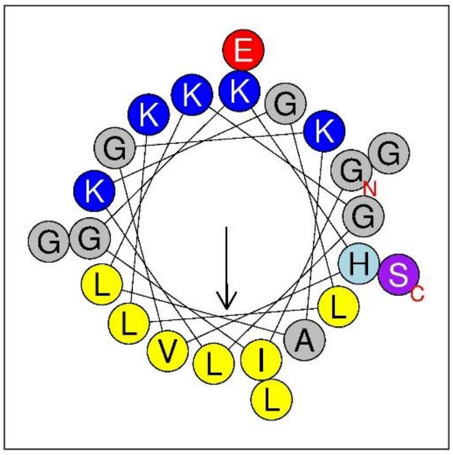

**Hydrophobicity** <H>	**Polar residues + GLY (n / %)**	**Nonpolar residues (n / %)**
0.275	15 / 65.22	8 / 34.78
**Hydrophobic moment** <μH>	**Uncharged residues + GLY**	**Aromatic residues**
0.596	HIS 1, SER 1, GLY 7	
**Net charge** z	**Charged residues**	**Special residues**
4	LYS 5, GLU 1,	CYS 0, PRO 0
**Hydrophobic face :** L A I L L V L L		

Figure S3. HeliQuest online server calculation result (https://heliquest.ipmc.cnrs.fr/). GIGKHVGKALKGLKGLLKGLGES adepantin-1 sequence is input, and output is hydrophobicity, 2D hydrophobic moment and other peptide properties. Eisenberg scale was used for calculation of hydrophobic properties.

B) Peptides in AA-1 and AA-2 simulations

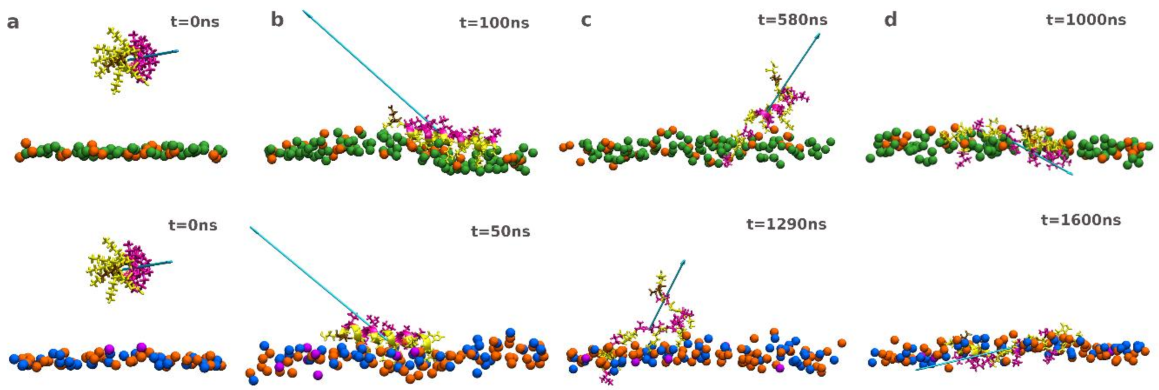

Figure S4. Representation of the single adeptantin-1 interacting with Gram-negative (on the top) and Gram-positive (on the bottom) bacterial membrane, where snapshot (**a**) is initial state, (**b**) shows electrostatic bonding of charged residues with upper leaflet lipids, (**c**) depicts peptide conformation change, and (**d**) represents the final state with hydrophobic residues inserted in the membrane interior. The 3D-HM vector is depicted as a cyan arrow, where length of the arrow corresponds to the 3D-HM value (Table S3). Peptides are depicted as ribbons and lines, with hydrophobic residues in magenta and polar and charged residues in yellow. The upper leaflet of the membrane is represented by beads for P atoms that are shown in green for POPE lipids, orange for POPG lipids, blue for Lys-PG lipids, and violet for PVCL2 lipids. For clarity, other atoms and molecules of the membranes, as well as water molecules and ions, are removed.

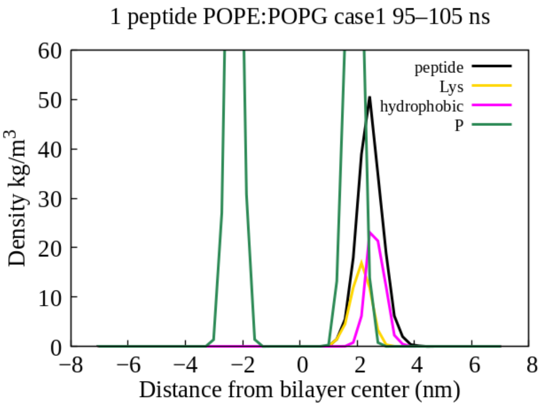

(**a**)

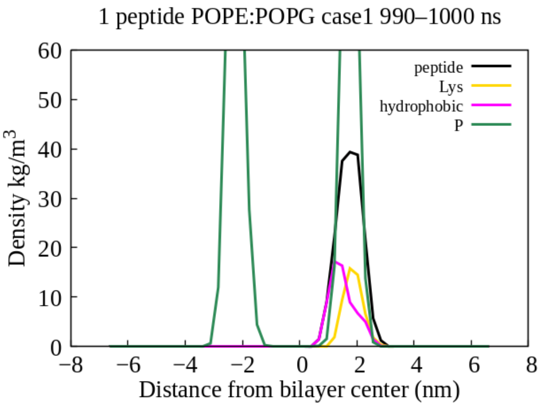

(**b**)

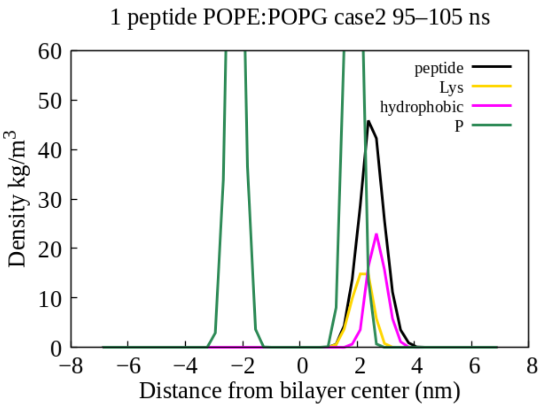

(**c**)

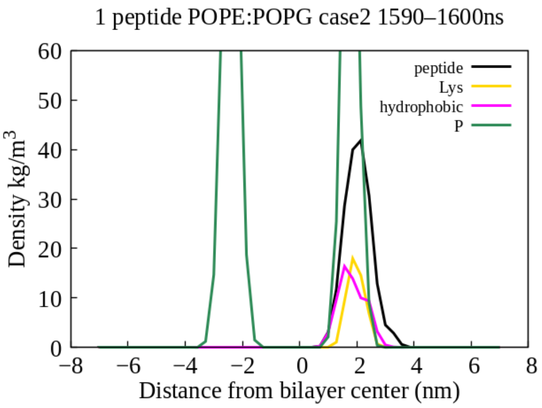

(**d**)

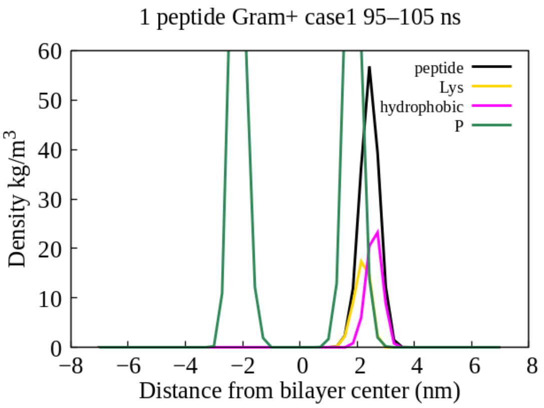

(**e**)

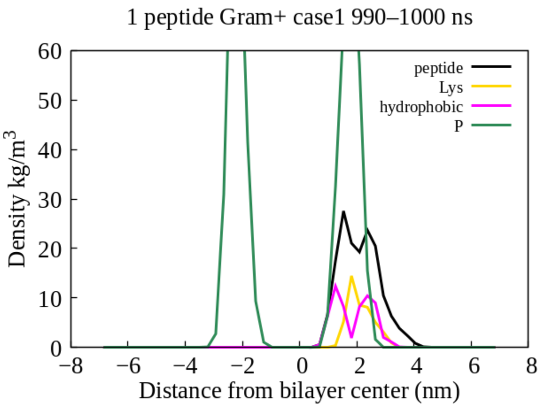

(**f**)

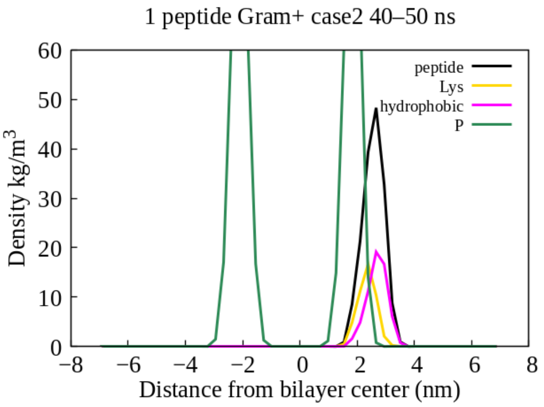

(**g**)

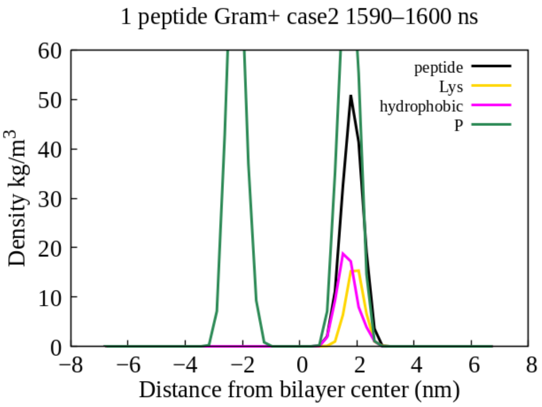

(**h**)

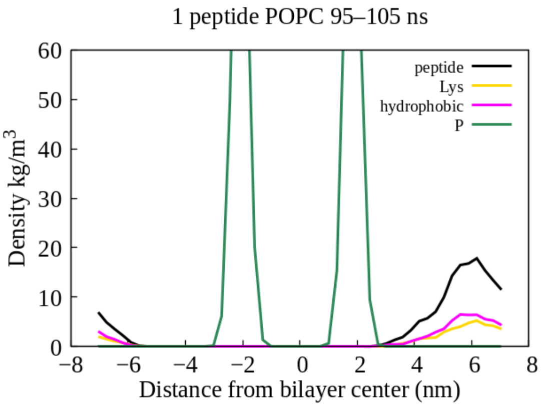

(**i**)

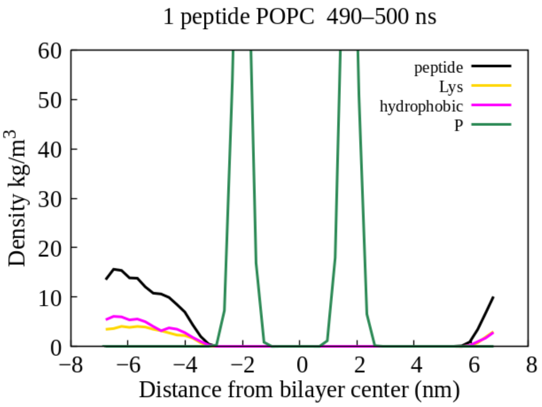

(**j**)

Figure S5. Density profiles calculated with Gromacs density tool of hydrophobic (magenta) and positively charged LYS residues (yellow) in AA-1 simulations, averaged over denoted time intervals. Left (**a**, **c**, **e**, **g**): At early stages of interaction of the peptide with the membrane, peptide is oriented in such a way that positively charged Lysine residues are closer to the anionic upper leaflet surface than hydrophobic residues (except for POPC membrane (**i**), where no binding occurs), supporting the role of electrostatic interactions in initial binding. Right (**b**, **d**, **f**, **h**): At later stages hydrophobic residues are closer to the central plane of the membrane revealing the turnover of the peptide over time (except for POPC membrane (**j**), where no binding occurs) and importance of hydrophobic interactions with carbohydrate lipid chains after initial electrostatic binding.

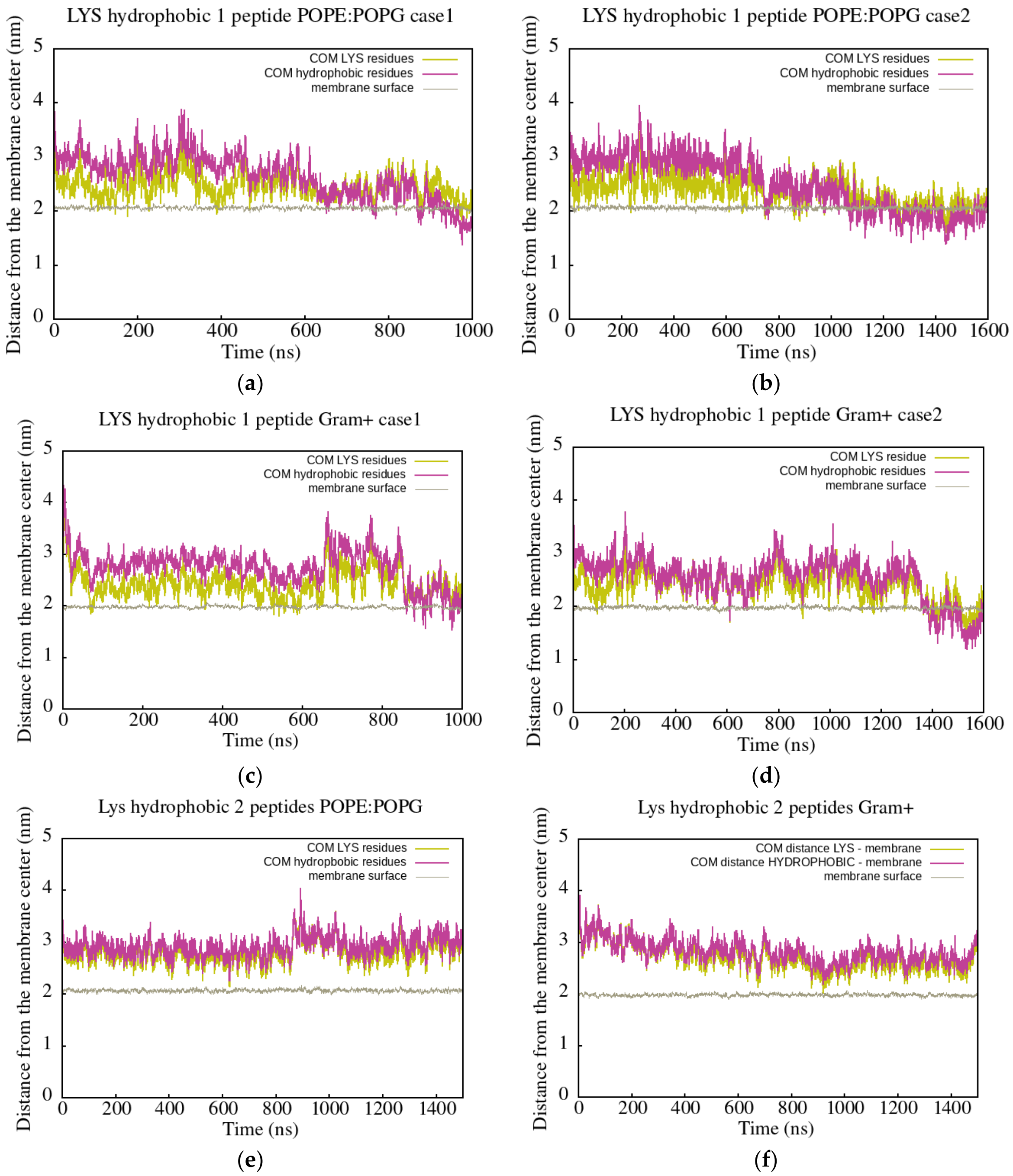

Figure S6. Distances from the membrane center to the COM of LYS (yellow line) and hydrophobic (magenta line) residues as a function of time for simulations of one or two peptide with Gram-negative and Gram-positive membranes. The first row Gram-negative AA-1 simulations (**a**, **b**), the second row Gram-positive AA-1 simulations (**c**, **d**), while the third row are results for AA-2 simulations, Gram-negative on the left (**e**) and Gram-positive on the right (**f**). Figures of one peptide simulations show that while LYS residues initially come into contact with the membrane surface, the positions of the hydrophilic LYS residues and hydrophobic residues shift over time, with the hydrophobic residues being placed closer to the bacterial membrane environment. Figure of two peptide simulations (**e**, **f**) show partitioning of hydrophobic and hydrophilic residues, but not the inversion of it during simulation time.

Table S3. 3DHM vector modules and angle between vector and +z axis at chosen states corresponding to those described in Figure 1 in the main article. Two dielectric constants are used for calculations: 78.5 for peptide surrounded by solution in initial state (t = 0) and 20.0 for peptide in solution-membrane interface. Early stages of interaction of the peptide with the membrane (Figure 1a) are electrostatic in nature and causes rotation of peptide's charged residues towards the membrane and consequently large HM vector module followed by orientation of the HM vector away from the membrane (angle < 90°). After initial electrostatic binding, other interactions cause peptide to rotate and change its conformations causing lowering of the HM vector angle and module (Figure 1b). Finally, peptide orients its hydrophobic residues towards the membrane which is also followed by orientation of the HM vector towards the membrane (angle > 90°) (Figure 1c).

Simulation case	t (ns)	Dielectric constant	3DHM vector module (AkT/e)	Angle between 3DHM vector and +z-axis (°)
**AA-1a POPE:POPG**	0	78.5	15.6	82.4
	100	20.0	60.7	50.0
	580	20.0	35.4	37.7
	1000	20.0	29.0	112.0
**AA-1b Gram+**	0	78.5	15.5	82.6
	50	20.0	54.2	48.6
	1290	20.0	28.4	35.1
	1600	20.0	33.2	102.4

C) Peptides in AA-12 simulations

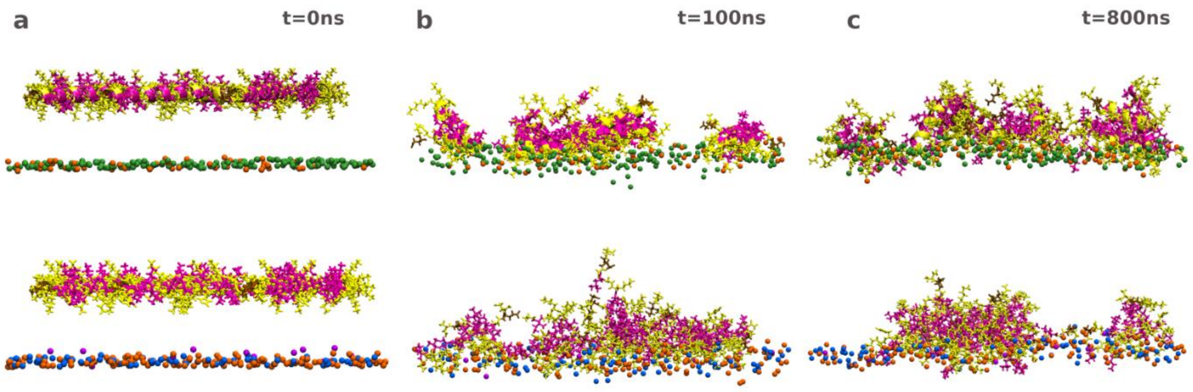

Figure S7. Snapshots of spatial distribution of hydrophobic (magenta) and hydrophilic (yellow) residues at various times in AA12 simulations (up: POPE: POPG, down: Gram+). Only the upper leaflets of membrane are shown, represented with P atoms (POPE green, POPG orange, Lys-PG blue, PVCL2 violet). (**a**) initial state, (**b**) electrostatic bonding of charged residues with upper leaflet lipids, (**c**) hydrophobic residues start inserting into membrane interior.

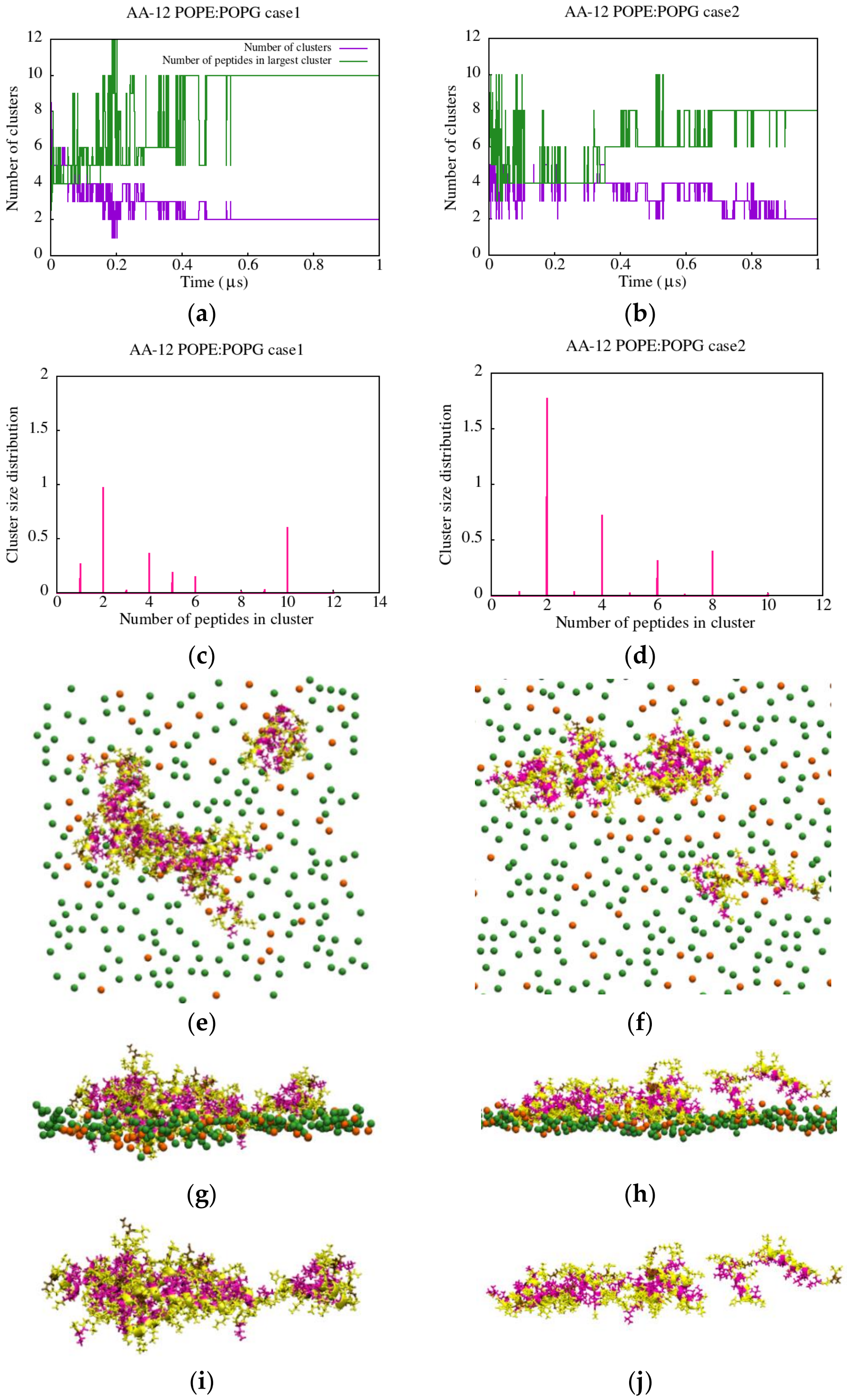

Figure S8. Cluster analysis calculated by clust Gromacs utility and cluster snapshots for AA-12 POPE:POPG simulations. The results for case 1 simulations (1 μs) are shown on the left, and the results for case 2 (1 μs) are on the right. The first row (**a**, **b**) show the number of clusters (in purple) and maximal size cluster (in green) as a function of simulation time. The second row (**c**, **d**) show cluster size distribution depending on the number of peptides in cluster. The last three rows (**e**–**j**) represent the snapshots of the clusters (top and side view) at the 1 μs simulation time. The results show varying number of clusters, where two are formed at the simulation end. The cluster size distributions show appearance of the various cluster sizes and two-peptide aggregate as the most probable. The snapshots show that at the simulation time of 1 μs the two observed clusters have two and ten peptides, respectively. The snapshots also indicate strong separation of hydrophilic and hydrophobic residues, where clusters have a hydrophobic core and hydrophilic surface which then are partly exposed to the membrane environment. Peptides are shown in magenta for hydrophobic and in yellow for hydrophilic residues.

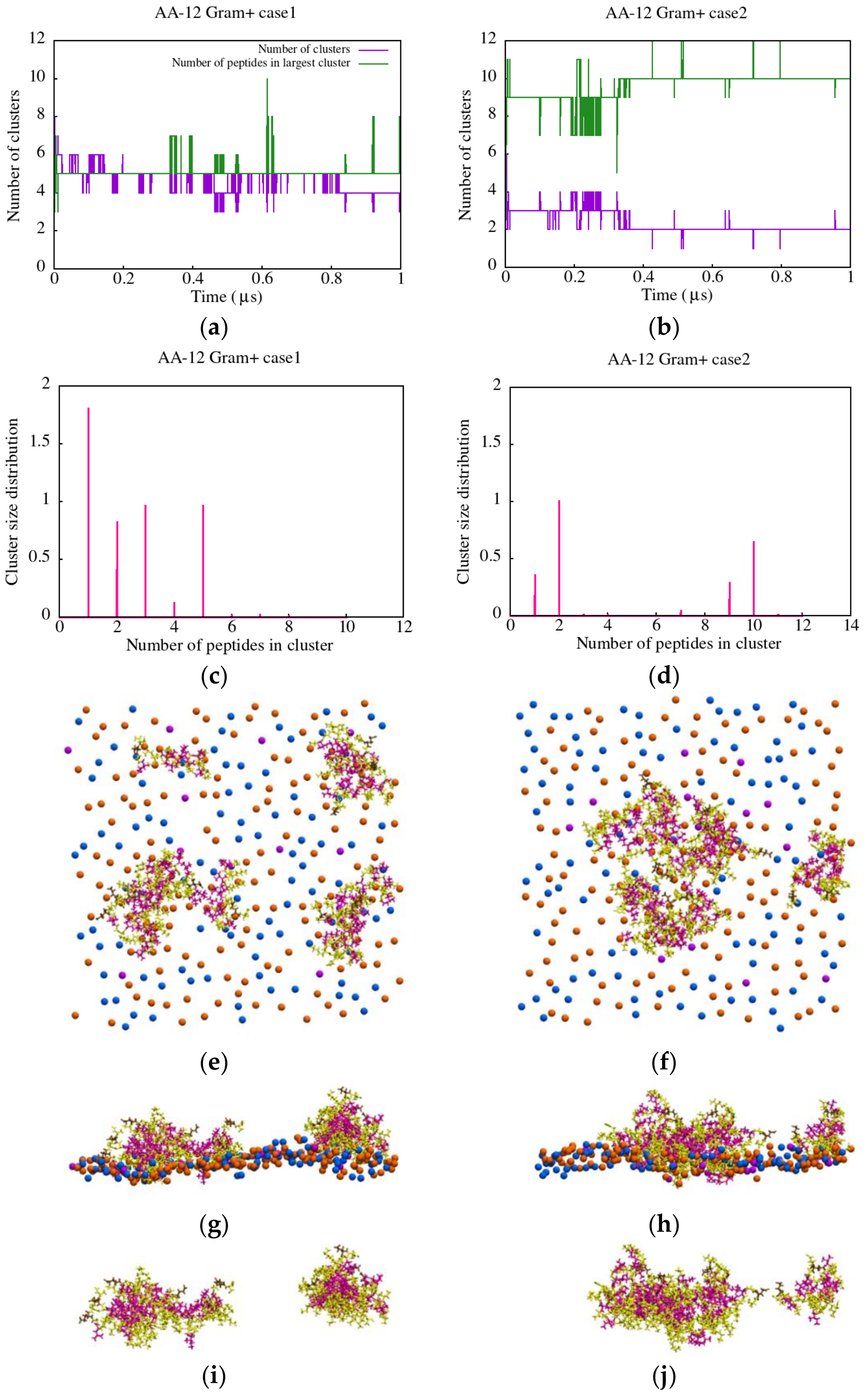

Figure S9. Cluster analysis calculated by clust Gromacs utility and cluster snapshots for AA-12 Gram+ simulations. The results for case1 simulations (1 μs) are shown on the left and the results for case2 (1 μs) are on the right. The first row (**a**, **b**) show the number of clusters (in purple) and maximal size cluster (in green) as a function of simulation time. The second row (**c**, **d**) show cluster size distribution depending on the number of peptides in cluster. The last three rows (**e**–**j**) represent the snapshots of the clusters (top and side view) at the 1 μs simulation time. The results show varying number of clusters, small size clusters in simulations case1 and one large and other small size cluster in simulation case2. The snapshots indicate, similar to Gram-membrane results, strong separation of hydrophilic and hydrophobic residues, where clusters have a hydrophobic core and hydrophilic surface which then are partly exposed to the membrane environment. Peptides are shown in magenta for hydrophobic and in yellow for hydrophilic residues.

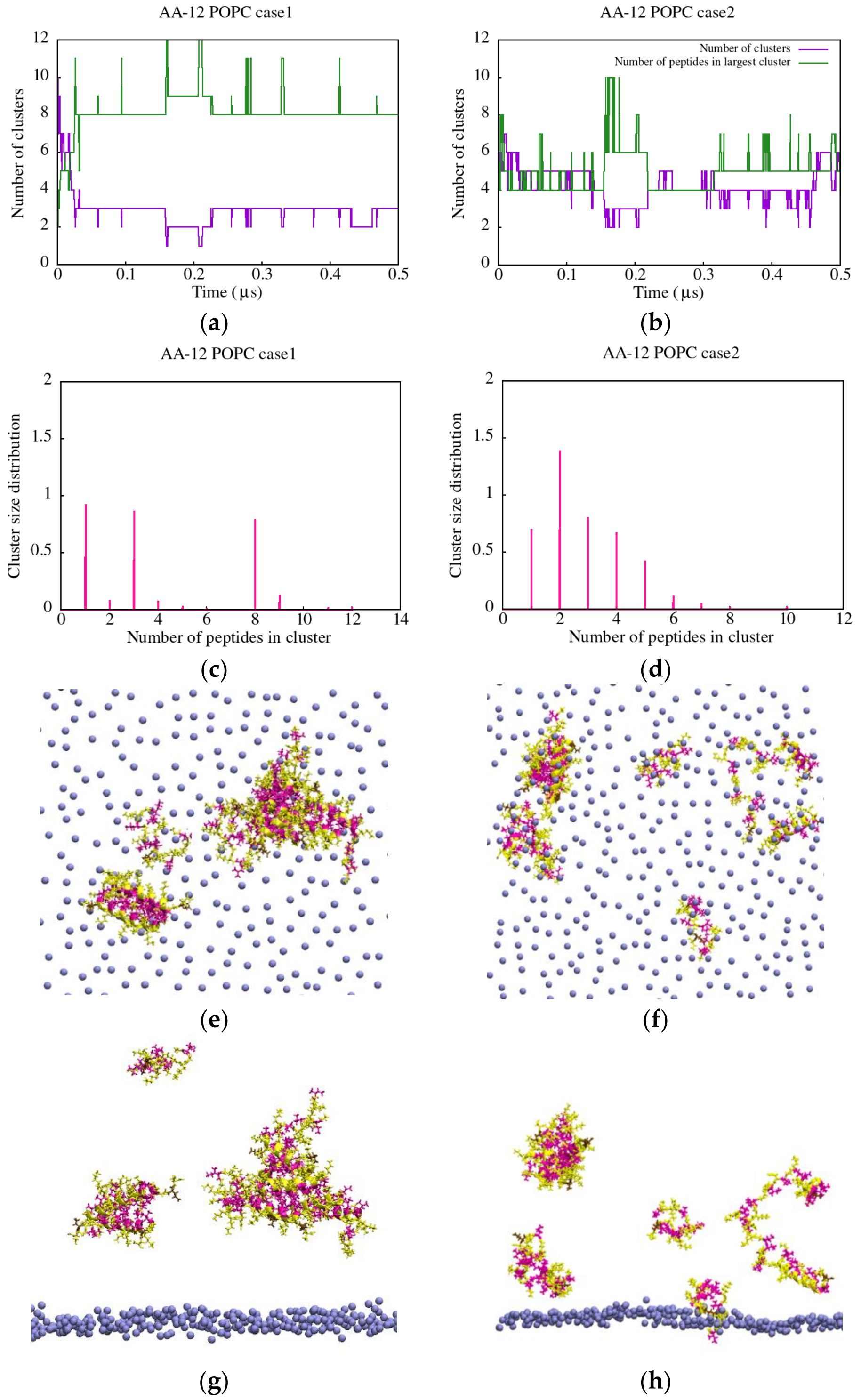

Figure S10. Cluster analysis calculated by clust Gromacs utility and cluster snapshots for AA-12 POPC simulations. The results for case 1 simulation (0.5 μs) are shown on the left, and the results for case 2 (0.5 μs) are on the right. The first row (**a**, **b**) show the number of clusters (in purple) and maximal size cluster (in green) as a function of simulation time. The second row (**c**, **d**) show cluster size distribution depending on the number of peptides in a cluster. The last three rows (**e**–**j**) represent the snapshots of the clusters (top and side view) at the 0.5 μs simulation time. The results show a varying number of clusters compared to cluster distribution for the cases of bacterial membranes. The cluster size distributions show that clusters of all sizes are present, and overall, clusters with a smaller number of peptides are more probable. The snapshots indicate that clusters are formed in water, as no or rare binding to POPC membrane is observed, and that a strong separation of hydrophilic and hydrophobic residues is also present. Peptides are shown in magenta for hydrophobic and in yellow for hydrophilic residues.

D) Peptides in CG and CG2AA simulations

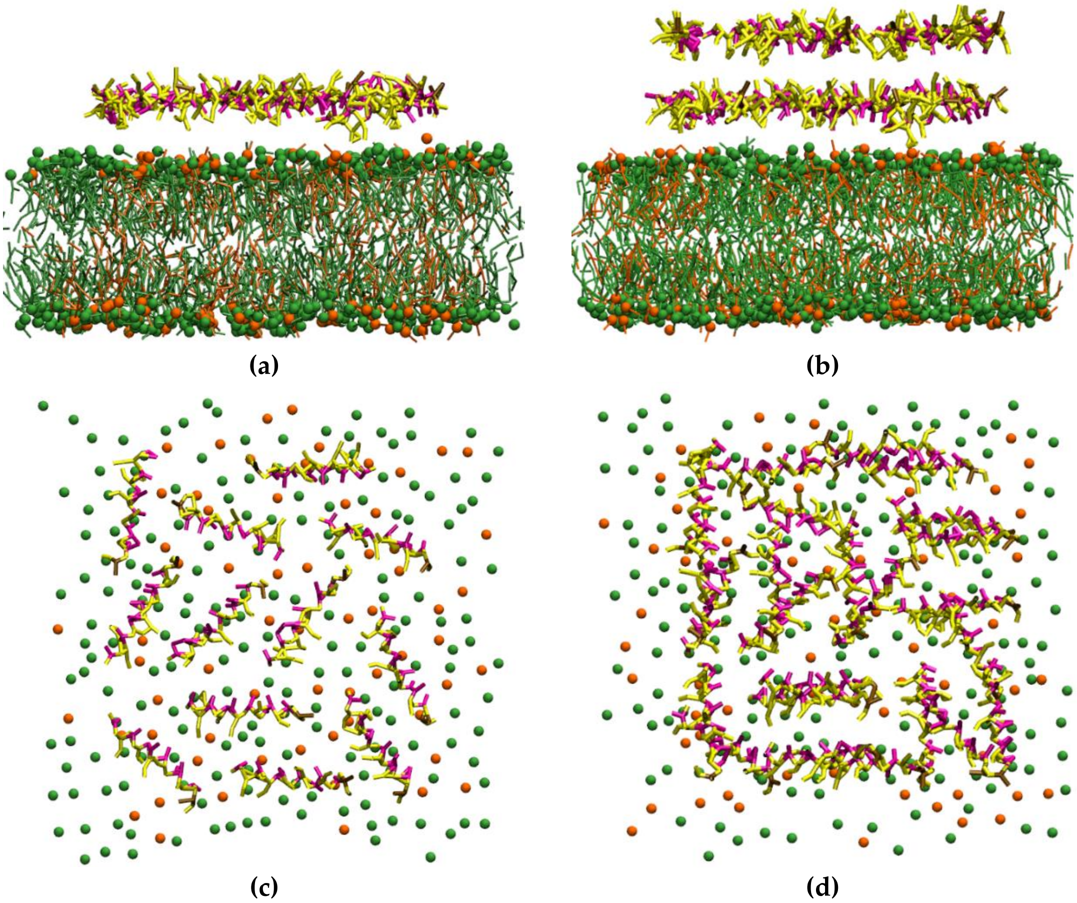

Figure S11. Initial configurations (side and top views) in CG simulations, on the left (**a**, **c**) case with twelve peptides and on the right (**b**, **d**) case with twenty-four peptides. Peptides are shown as sticks, in magenta for hydrophobic and in yellow for hydrophilic residues. The membrane is represented by P atom beads in green for POPE lipids and orange for POPG lipids. Other atoms and molecules of the membranes, as well as water molecules and ions are removed for clarity.

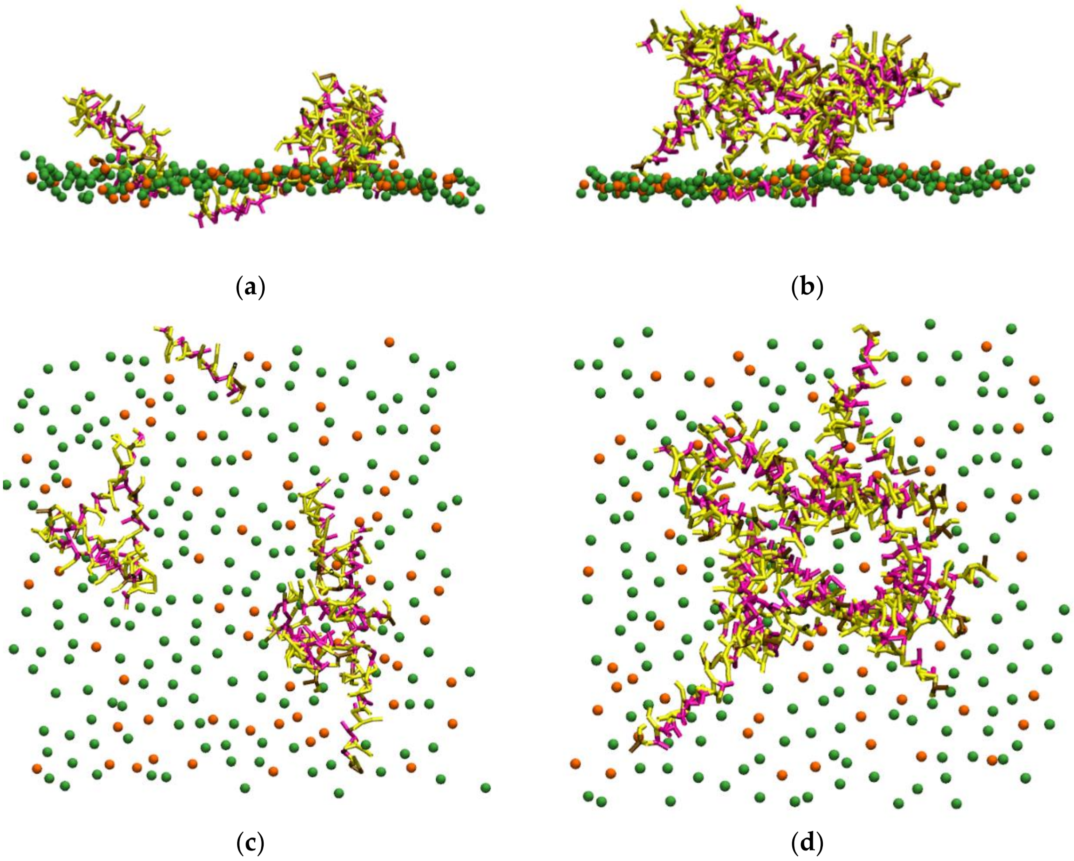

Figure S12. Snapshots represent formation of large aggregates at the early simulation time (~15 ns) in CG-12 (**a**, **c**) and CG-24 (**b**, **d**) simulations. In top and side views, peptides are shown as sticks, in magenta for hydrophobic and in yellow for hydrophilic residues. The membrane is represented by P atom beads in green for POPE lipids and orange for POPG lipids. Other atoms and molecules of the membranes, as well as water molecules and ions are removed for clarity.

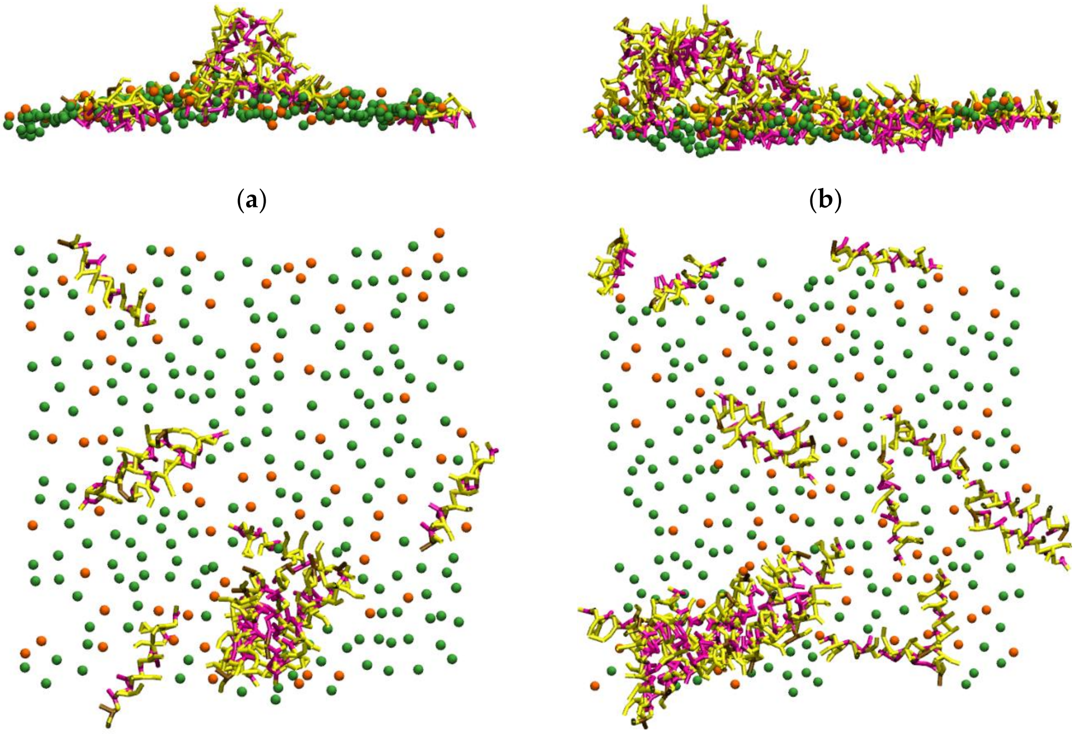

(**a**) (**b**)

(**c**) (**d**)

Figure S13. Snapshots represent aggregates at the end of CG-12 (t = 25 μs) (**a**, **c**) and CG-24 (t = 42.5 μs) (**b**, **d**) simulations, where the peptide migration is visible by comparing the snapshots in Figure S12. In top and side views, peptides are shown as sticks, in magenta for hydrophobic and in yellow for hydrophilic residues. The membrane is represented by P atom beads in green for POPE lipids and orange for POPG lipids. Other atoms and molecules of the membranes, as well as water molecules and ions are removed for clarity.

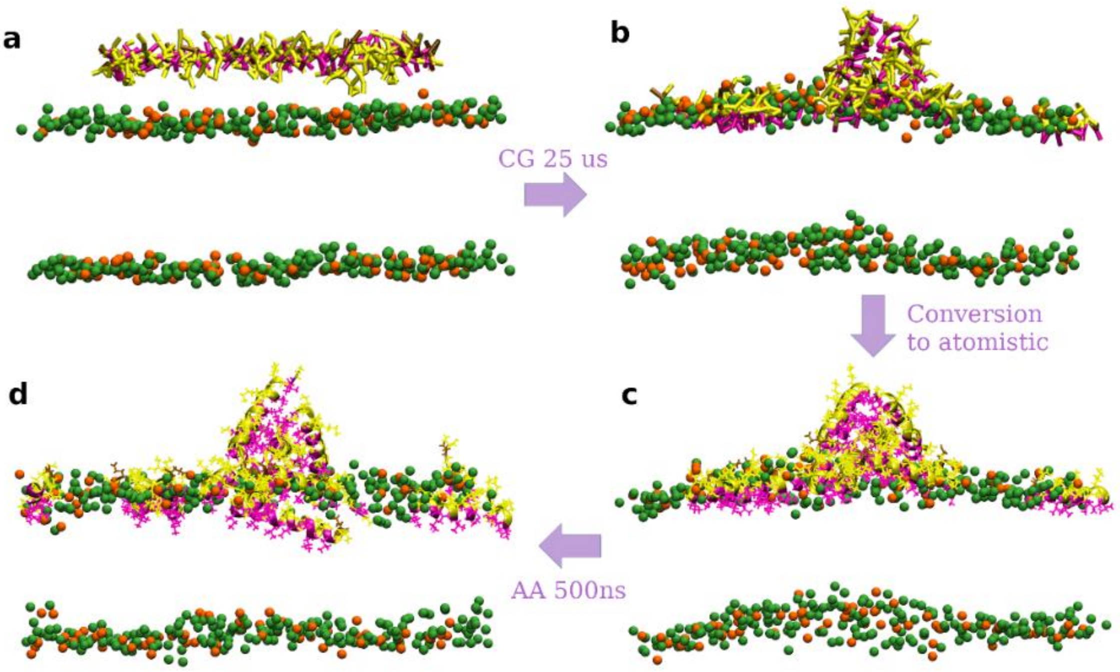

Figure S14. Representation of twelve adepantins interacting with Gram-negative bacterial membranes, where snapshot (**a**) represents the initial state for CG simulation, snapshot (**b**) represents final state of CG simulation at the simulation time of 25 µs, (**c**) depicts mapping CG system to AA system and snapshot (**d**) final state of CG2AA simulation at 500 ns simulation time. Peptides are represented as ribbons and lines, with hydrophobic residues colored magenta and polar and charged residues colored yellow. The membrane is represented by P atom beads in green for POPE lipids and orange for POPG lipids. Other atoms and molecules of the membranes, as well as water molecules and ions are removed for clarity.

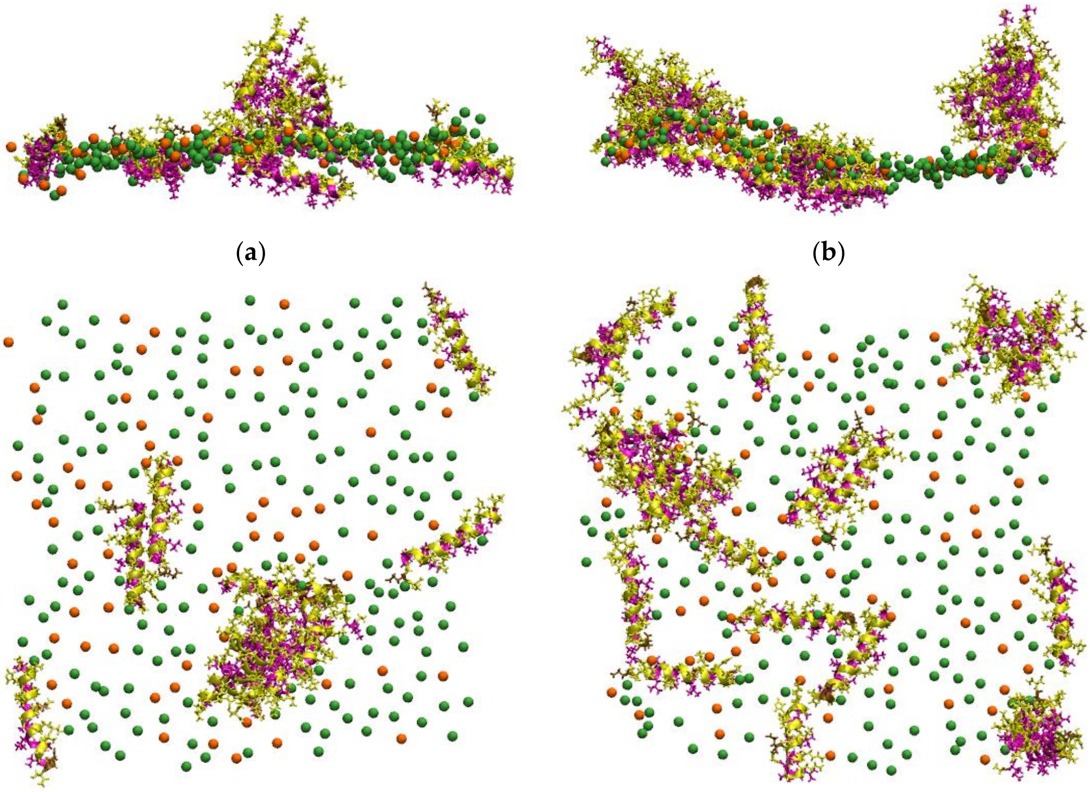

(**a**) (**b**) (**c**) (**d**)

Figure S15. Snapshots are taken from the last configurations in CG2AA simulations (500 ns), on the right the case with twelve, and on the right the case twenty-four peptides. Peptides are represented as ribbons and lines, with hydrophobic residues colored magenta and polar and charged residues colored yellow. The membrane is represented by P atom beads in green for POPE lipids and orange for POPG lipids. Other atoms and molecules of the membranes, as well as water molecules and ions are removed for clarity.

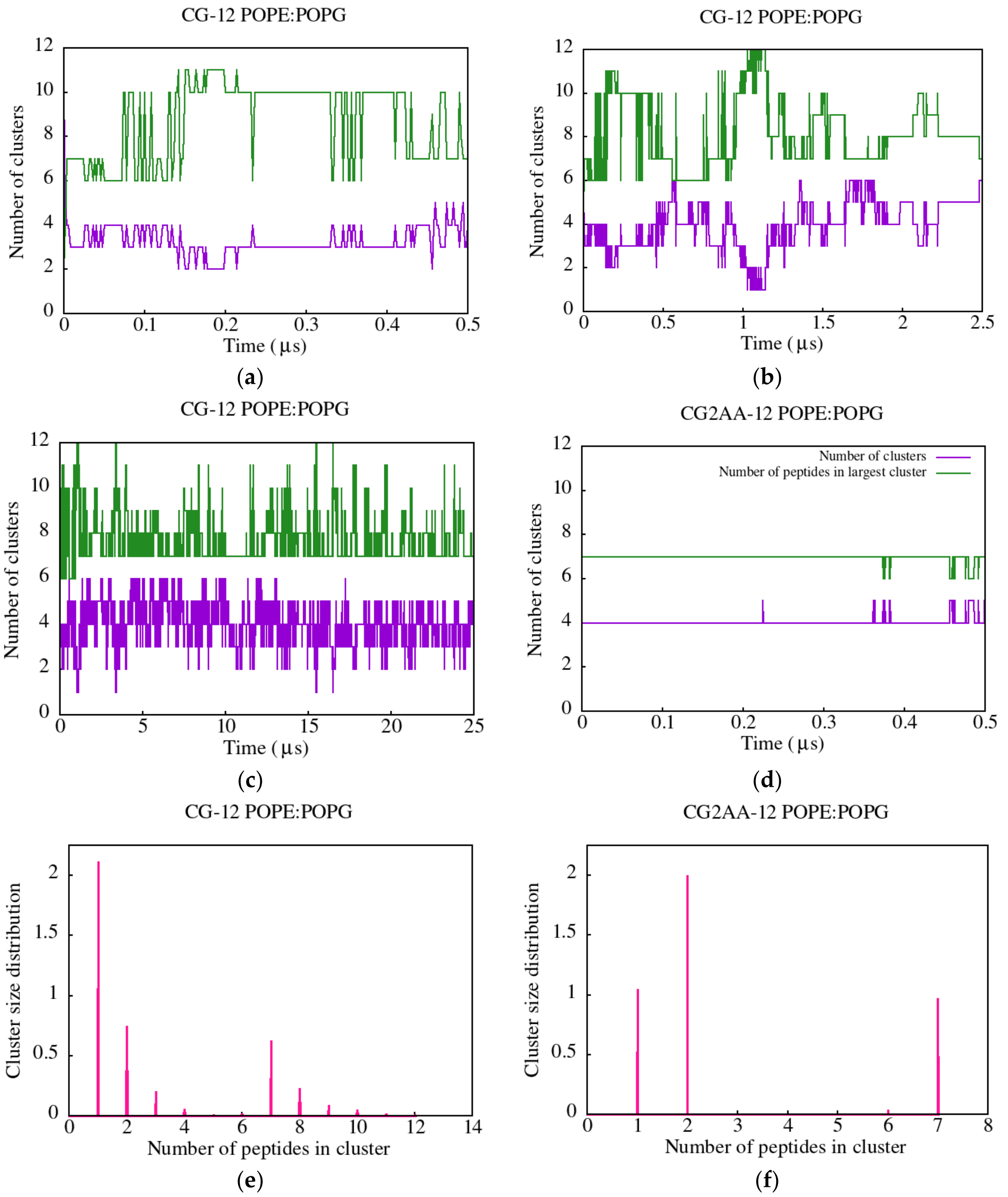

Figure S16. Cluster analysis calculated by clust Gromacs utility. The results for CG-12 simulations are shown in (**a**, **b**, **c**) and (**e**), while the results for CG2AA-12 simulations are shown in (**d**) and (**f**). The two upper rows show the number of clusters (in purple) and maximal size cluster (in green) as a function of simulation time, with the first row representing the short simulation time (0.5 μs and 2.5 μs) of CG-12 simulations, and the second row shows the results for total simulations time (CG-12 25 μs and CG2AA-12 0.5 μs), while the third row shows cluster size distribution depending on the number of peptides in cluster. The results show more variable cluster distribution for CG than CG2AA, which is possible due to the force field, but also to the substantially longer simulations times in GG compared to CG2AA runs. However, it is noticeable that peptides stay mostly single or form two-peptides aggregates or are part of the larger associations. These findings are witnessed by the strong opposite correlation between the number of clusters and maximal cluster size, which indicate that one large and other small size clusters are formed. Also, we noticed the bimodal character of cluster size distributions, especially highlighted in CG2AA simulation results where only clusters with small size (one and two peptides) and large size (six and seven peptides) are observed.

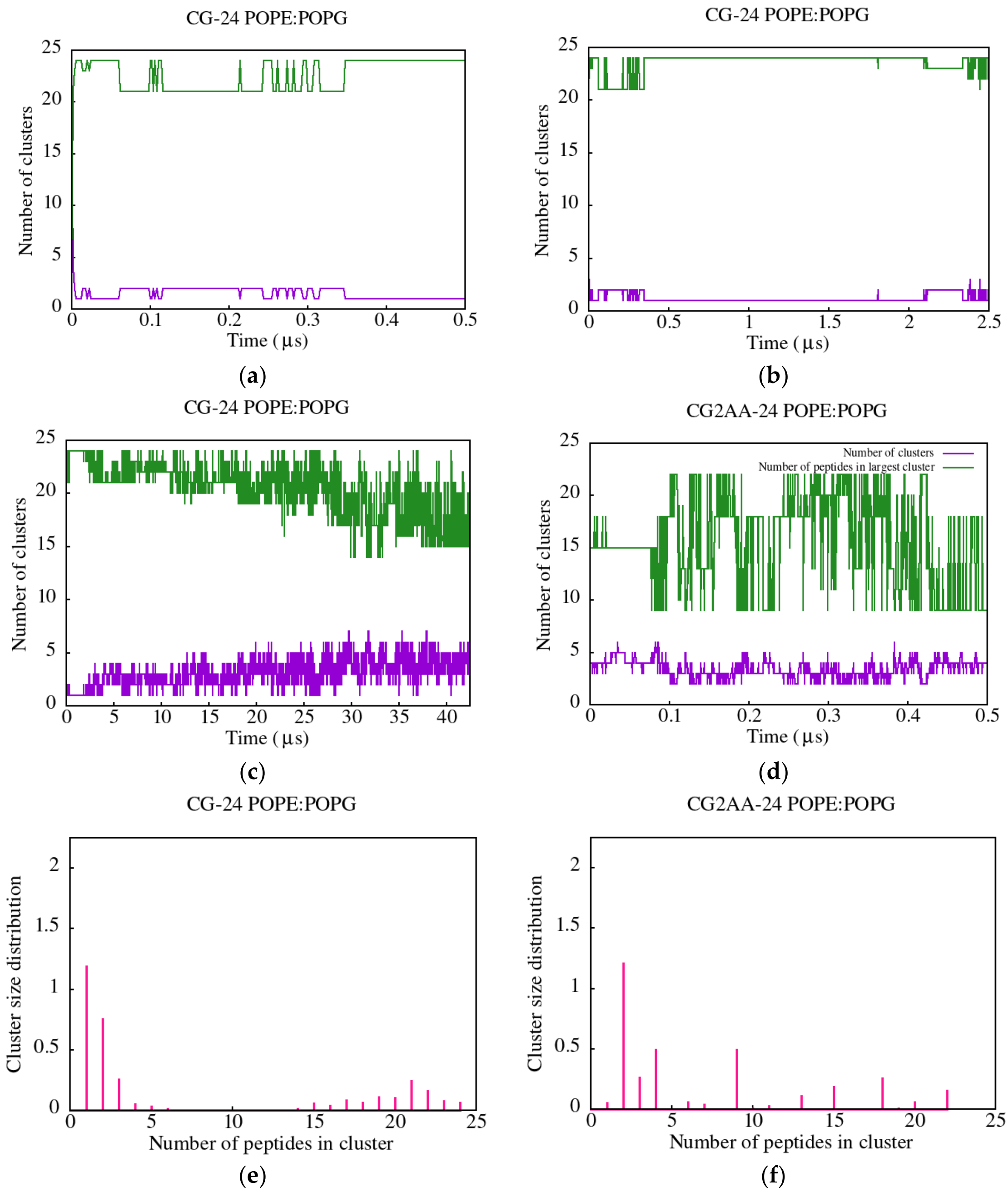

Figure S17. Cluster analysis calculated by clust Gromacs utility. The results for CG-24 simulations are shown in (**a**, **b**, **c**) and (**e**), while the results for CG2AA-24 simulations are shown in (**d**) and (**f**). The two upper rows show the number of clusters (in purple) and maximal size cluster (in green) as a function of simulation time, with the first row representing the short simulation time (0.5 μs and 2.5 μs) of CG-24 simulations, and the second row shows the results for total simulations time (CG-24 42.5 μs and CG2AA-24 0.5 μs), while the third row shows cluster size distribution depending on the number of peptides in cluster. In the CG-24 simulation, initially peptides group in one cluster, which is partially also due to the initial position of peptides (see Figure S11 b,d), but with the progress of simulation time, the number of clusters increases and stabilizes. Again, there is a strong opposite correlation between the number of clusters and maximal number of peptides in the cluster. The strong separation of small and larger size cluster in cluster size distributions are also visible, however only for the CG simulations. We may suppose that in the case of CG2AA-24 simulation, the number of peptides in one cluster varies more when a larger number of peptides are present at the same membrane size, just as an effect of the peptide's proximity and due to that the bimodal distribution is less visible compared to the case of CG2AA-12. It is worth noticing that in both CG2AA simulations the most probable clusters are the one with two peptides.

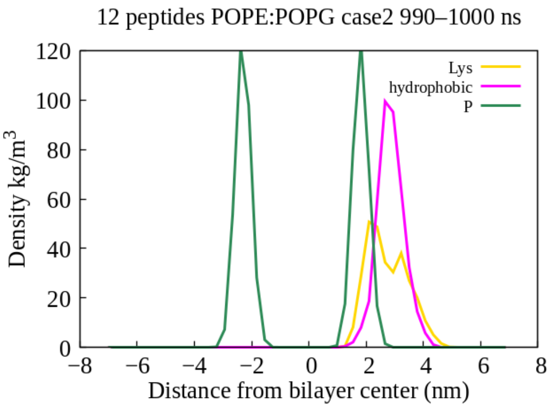

(**a**)

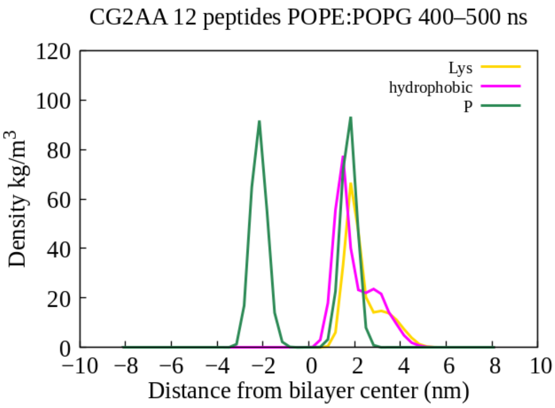

(**b**)

Figure S18. Density profiles calculated with Gromacs density tool of hydrophobic (magenta) and positively charged Lys residues (yellow), averaged over denoted time intervals, on the left AA-12 results, and on the right CG2AA-12 results. Over the course of AA-12 simulations (~1 µs), peptides forms clusters in which fraction of positively charged Lys residues stays closer to the membrane central plane than the hydrophobic residues, suggesting that insertion dynamics is slower than in case of one peptide (see Figures S5 a–d and S6 a,b). CG2AA-12 simulations results (calculated after 25 µs CG and 500 ns CG2AA simulations) reveal the same behavior as in case of single peptide: some of the hydrophobic residues are closer to the membrane central plane than charged Lysins, again revealing the turnover of some peptides in the clusters over time and importance of hydrophobic interactions with acyl chains in process of peptide insertions into membrane interior.

E) Membrane analysis

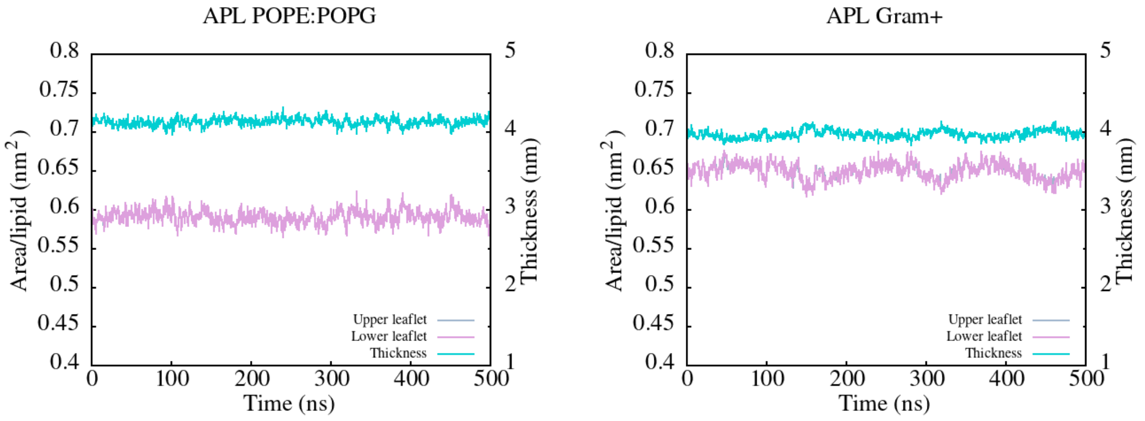

(**a**) (**b**)

Figure S19.1. Area per lipid and membrane thickness as a function of simulation time for pure membrane systems (AA-0 simulations). The results show that the Gram-positive membrane (**b**) exhibits slightly larger fluctuation compared to Gram-negative membrane (**a**). The simulation APL results agree well with the literature data as shown in Table S2. .

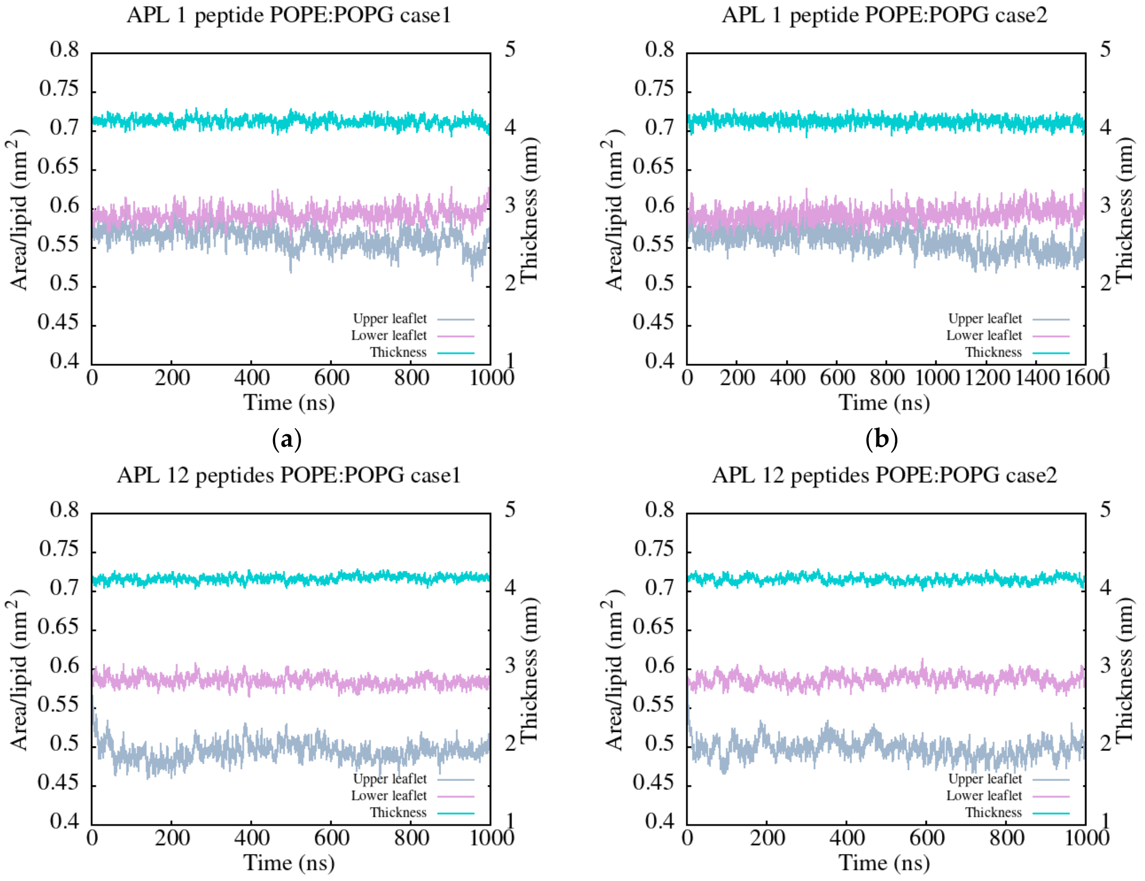

(**a**) (**b**) (**c**) (**d**)

Figure S19.2. Area per lipid and membrane thickness as a function of simulation time for AA simulations of Gram-negative membrane (POPE:POPG bilayer). The top row shows AA-1 simulations and bottom row AA-12 simulations (case1 is on the left, and case2 is on the right). The results show that for one-peptide simulations the difference in area per lipids between leaflets is very small but increases with the time, whereas in the case of twelve peptide simulations, this difference is larger, forms within the first 100 ns, and it remains stable over time. The membrane thickness shows small but noticeable changes in size compared to thickness of the solvated membrane system without the peptides, but it is also stable over time.

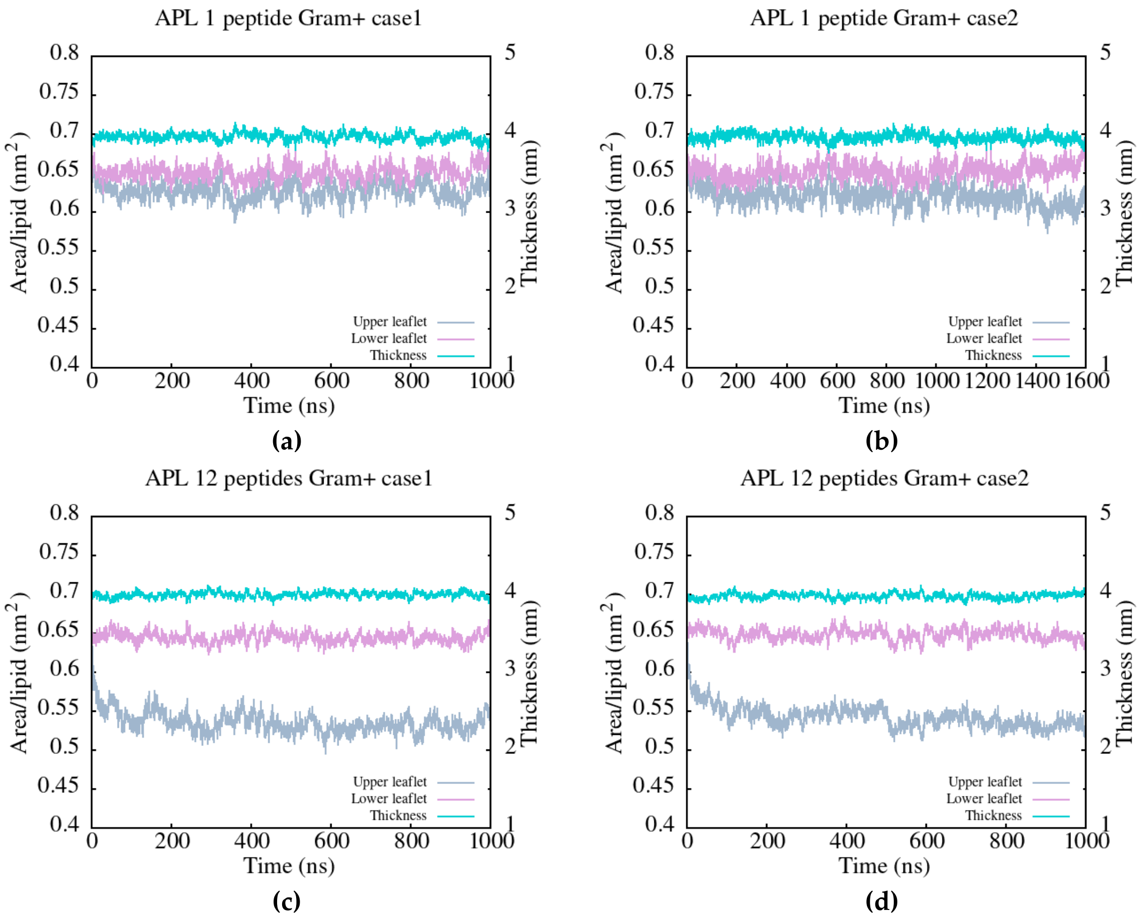

Figure S19.3. Area per lipid and membrane thickness as a function of simulation time for AA simulations of Gram-positive membrane (POPG:Lys-PG:PVCL bilayer). The top row shows AA-1 simulations and bottom row AA-12 simulations (case1 is on the left, and case2 is on the right). The Gram-positive membrane has more enhanced fluctuation of area per lipid compared to Gram-negative membrane, as illustrated for the solvated membranes without peptides in Figure S19.1, but similar behavior when observing the changes in the area per lipids induced by the peptides. Similar to Gram-negative membrane, membrane thickness is smaller than in membrane without the peptides and stable over time.

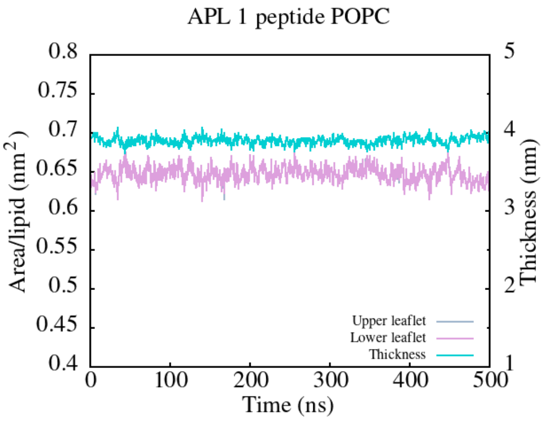

Figure S19.4. Area per lipid and membrane thickness as a function of simulation time for AA simulations of the neutral membrane (POPC bilayer). The AA-1 simulation results are presented, but the same behavior is observed in other simulation cases with POPC membrane. The peptides do not bind to the membrane, and the results are the same as for pure membrane without peptides (see Table S2).

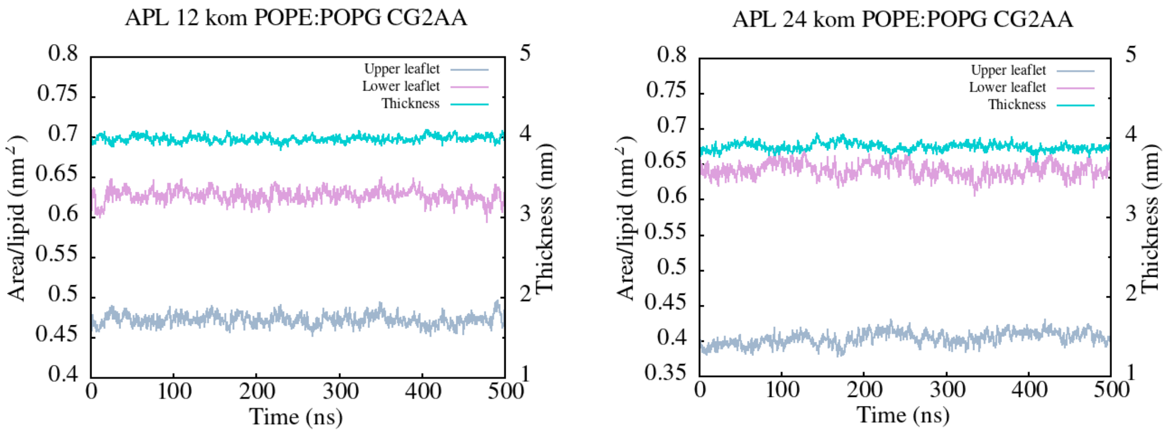

(**a**) (**b**)

Figure S19.5. Area per lipid and membrane thickness for CG2AA simulations of Gram-negative membrane as a function of simulation time (POPE:POPG bilayer). The results of CG2AA-12 simulation are on the left and CG2AA-24 simulation results are on the right. The results show that for the 24-peptide simulations compared to 12-peptide simulation, thickness is smaller, and difference of the upper leaflet APL in CG2AA-24 is bigger than CG2AA-12 and smaller for the lower leaflet, both compared to pure membrane (Figure S19.1a), which results in the larger overall difference between the leaflets for the CG2AA-24 case. However, in both simulations, all properties are stable over time.

Table S4. Area per lipid, total area of lipids and membrane thickness averages over last 100 ns of simulation time as calculated by APL@voro. ("up" – upper leaflet, "down"—lower leaflet).

		Area per lipid (nm^2)				Thickness	Total area of lipids (nm^2)		
		**Lipid**	**up**	**down**	**ratio**	**(nm)**	**up**	**down**	**ratio**
**AA-0**	Gram−	PE:PG	0.59	0.59	1	4.13	75.63	75.63	1
**AA-0**	Gram+	PG	0.65	0.64	1	3.97	83.15	83.15	1
		LPG	0.64	0.64	1				
		PVCL2	0.77	0.77	1				
		all lipids	0.65	0.65	1				
**AA-1a**	Gram−	PE:PG	0.56	0.60	0.93	4.09	70.64	76.40	0.92
**AA-1b**		PE:PG	0.54	0.59	0.92	4.10	69.68	76.21	0.91
**AA-1a**	Gram+	PG	0.61	0.65	0.94	3.97	80.14	83.9	0.96
		Lys-PG	0.62	0.64	0.97				
		PVCL2	0.75	0.76	0.99				
		all lipids	0.621	0.652	0.952				
**AA-1b**		PG	0.59	0.66	0.89	3.91	77.94	84.89	0.92
		Lys-PG	0.61	0.66	0.92				
		PVCL2	0.75	0.74	1.00				
		all lipids	0.606	0.664	0.912				
**AA-12a**	Gram−	PE:PG	0.50	0.58	0.86	4.17	128.16	149.80	0.85
**AA-12b**		PE:PG	0.50	0.58	0.86	4.17	127.70	149.54	0.85
**AA-12a**	Gram+	PG	0.50	0.64	0.78	4.02	137.00	164.88	0.83
		Lys-PG	0.58	0.64	0.90				
		PVCL2	0.53	0.75	0.71				
		all lipids	0.531	0.646	0.823				
**AA-12b**		PG	0.52	0.64	0.81	4.01	136.70	165.70	0.83
		Lys-PG	0.56	0.64	0.88				
		PVCL2	0.58	0.76	0.76				
		all lipids	0.538	0.646	0.833				
**CG2AA-12**	Gram−	PE:PG	0.47	0.63	0.74	3.98	121.20	160.60	0.75
**CG2AA-24**	Gram−	PE:PG	0.4	0.65	0.62	3.90	102.47	164.84	0.62

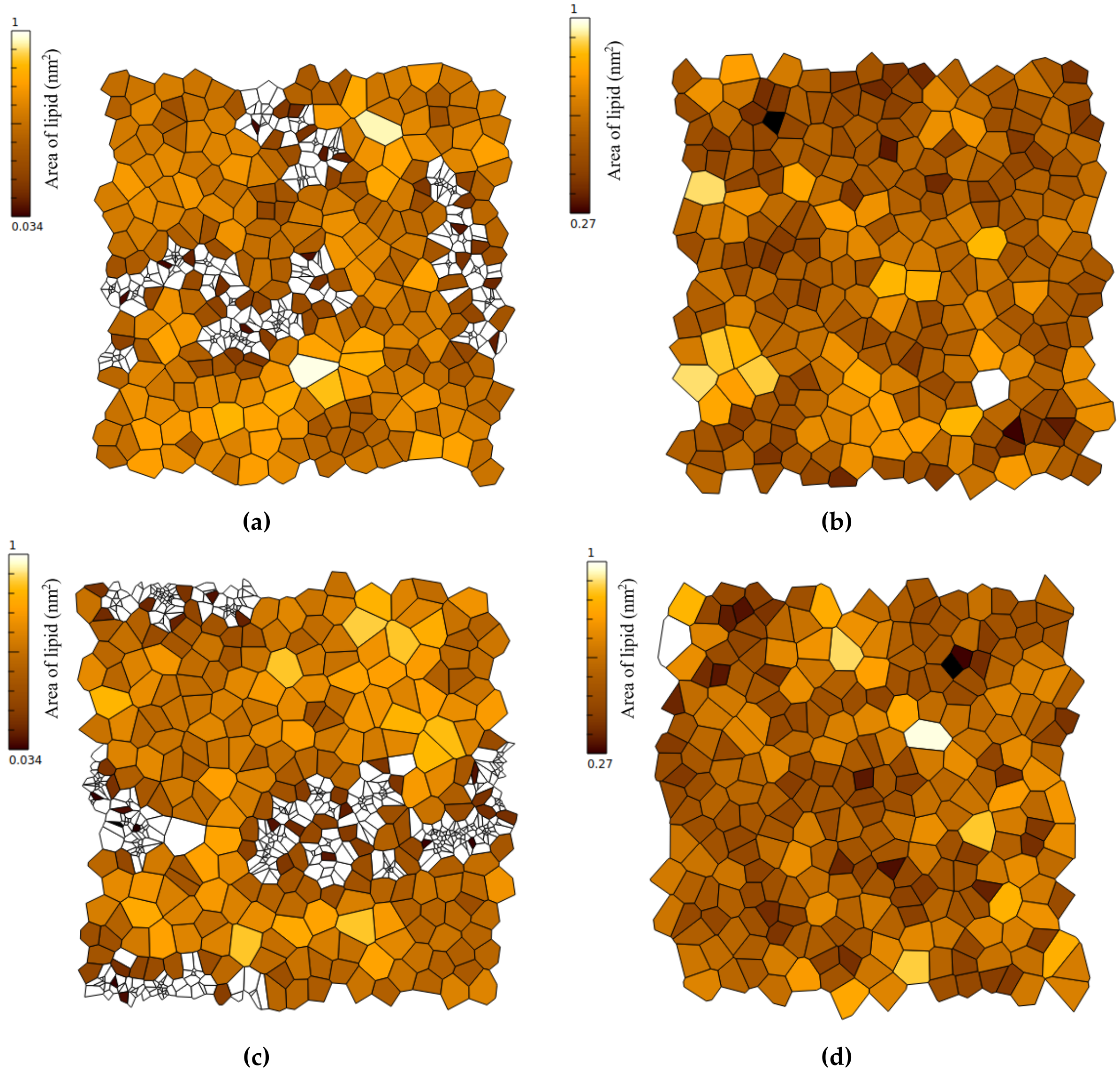

(a) (b) (c) (d)

Figure S20.1. Profiles of area of lipid calculated by APL@voro, based on Voronoi partitioning of the lipid surface for selected key atoms in lipid headgroups (phosphorus atoms) for the last frame (simulation time 1 μs) of AA-12 Gram- simulations. Upper leaflets (with proteins) are on the left (**a** and **c**), while lower leaflets are on the right (**b** and **d**). The case1 is in the top row and case2 is in the bottom row.

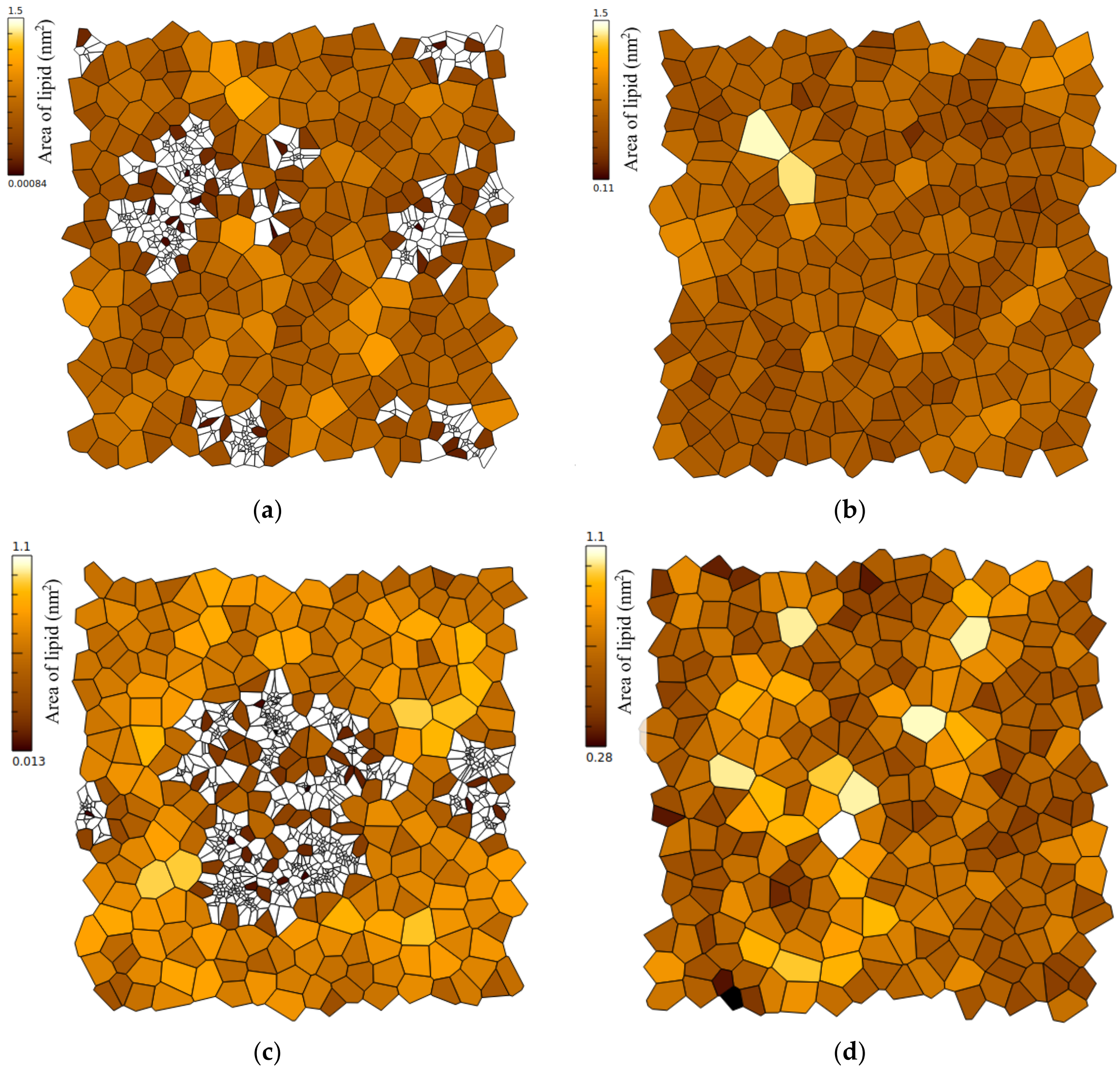

Figure S20.2. Profiles of area of lipid calculated by APL@voro, based on Voronoi partitioning of the lipid surface for selected key atoms in lipid headgroups (phosphorus atoms) for the last frame (simulation time 1 µs) of AA-12 Gram+ simulations. Upper leaflets (with proteins) are on the left (**a** and **c**), while lower leaflets are on the right (**b** and **d**). The case1 is in the top row and case2 is in the bottom row.

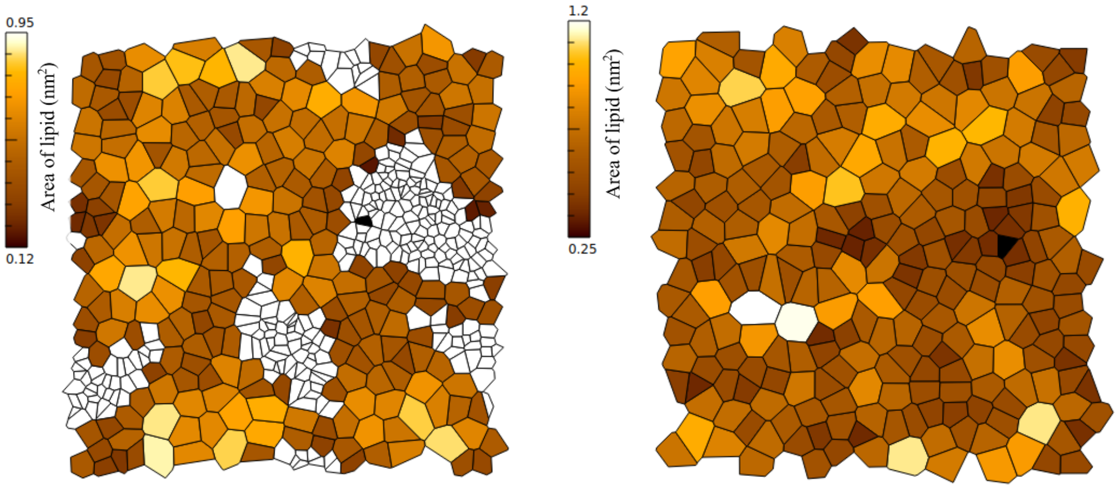

(**a**) (**b**)

Figure S20.3. Profiles of area of lipid calculated by APL@voro, based on Voronoi partitioning of the lipid surface for selected key atoms (phosphorus atoms) in lipid headgroups for the last frame (simulation time 25 μs) of CG-12 Gram- simulations. The upper leaflet (with proteins) is on the left (**a**), while lower leaflet is on the right (**b**).

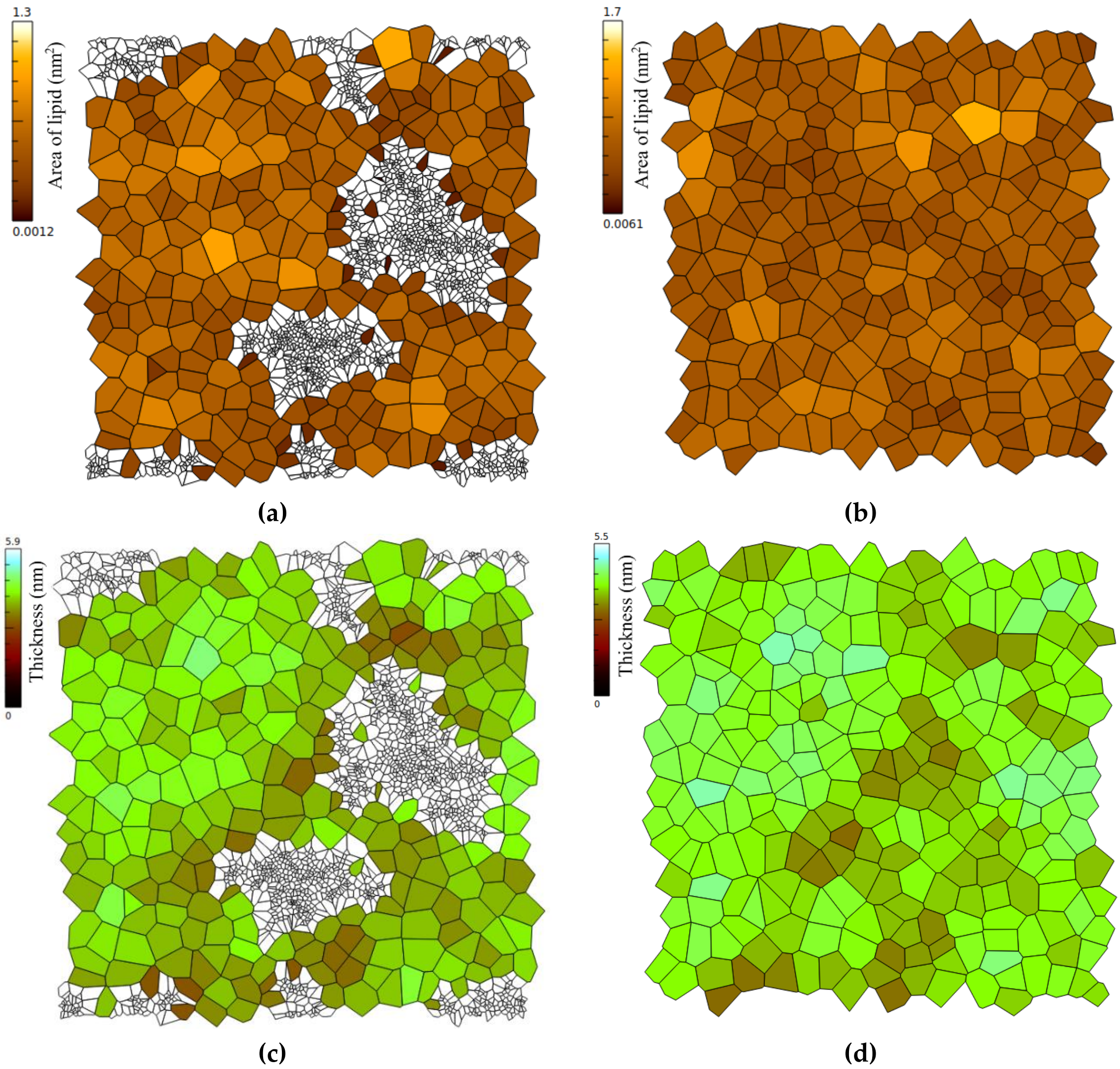

Figure S21.1. Profiles of area of lipid (top row) and membrane thickness (bottom row) calculated by APL@voro, based on Voronoi partitioning of the lipid surface for selected key atoms in lipid headgroups (phosphorus atoms) for the last frame of CG2AA-12 simulations (simulation time 500 ns). Upper leaflets (with proteins) are on the left (**a** and **c**) while lower leaflets are on the right (**b** and **d**).

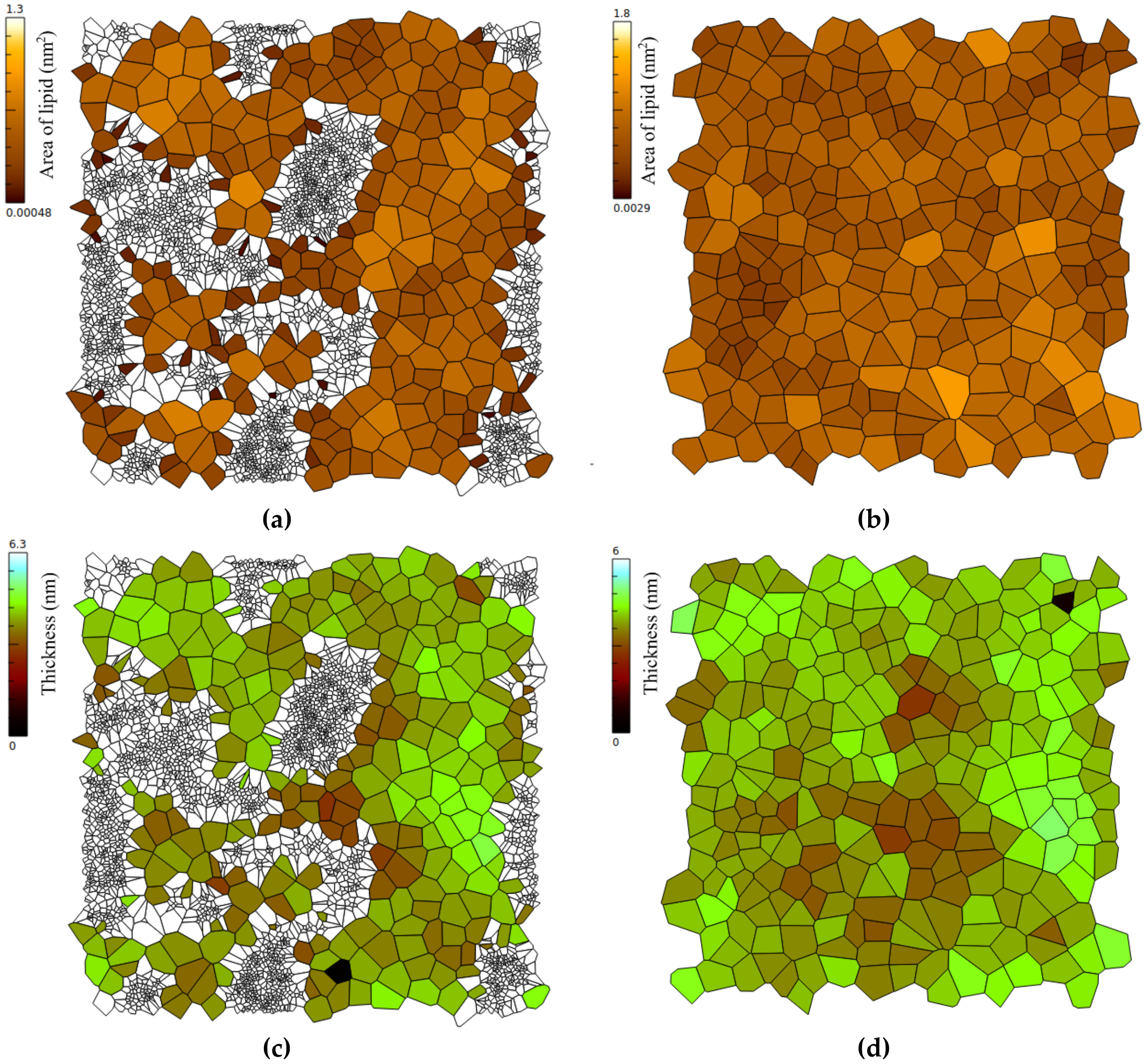

Figure S21.2. Profiles of area of lipid (top row) and membrane thickness (bottom row) calculated by APL@voro, based on Voronoi partitioning of the lipid surface for selected key atoms in lipid headgroups (phosphorus atoms) for the last frame (simulation time 500 ns) of CG2AA-24 simulations. Upper leaflets (with proteins) are on the left (**a** and **c**), while lower leaflets are on the right (**b** and **d**).

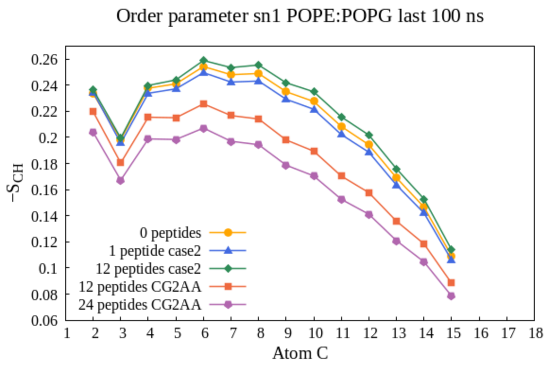

(**a**)

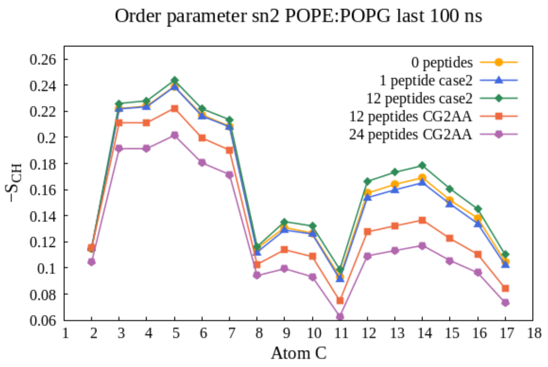

(**b**)

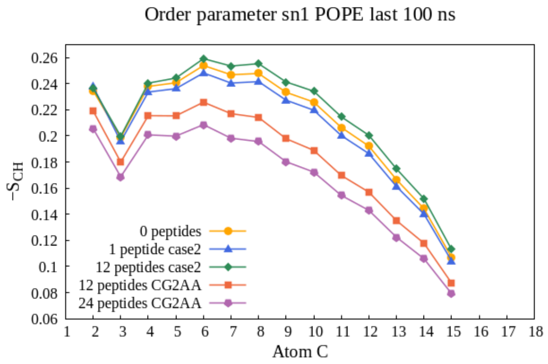

(**c**)

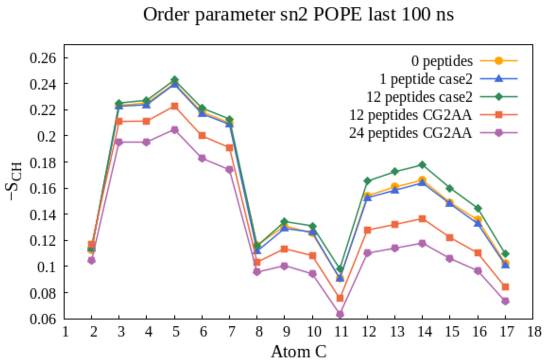

(**d**)

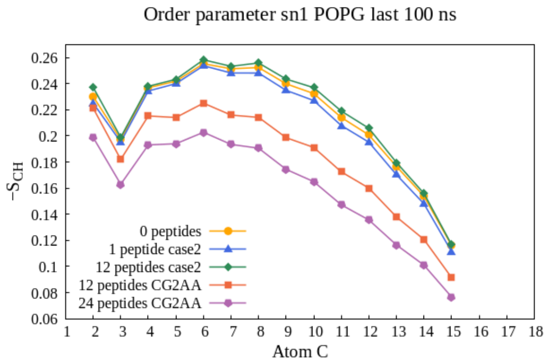

(**e**)

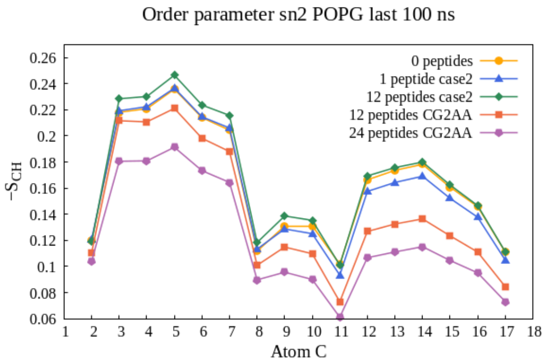

(**f**)

Figure S22.1. Order parameter $-S_{CH}$ for the acyl chains sn-1 (on the left) and sn-2 (on the right) as a function of the carbon atom index and calculated as averages over last 100 ns simulation time by order Gromacs utility. The legend is included in the graphs, and the results are shown for simulation cases with Gram- membrane (AA-0, AA-1b, AA-12b, CG2AA-12, CG2AA-24), with the results for POPE:POPG lipids in the first row (**a**, **b**), POPE lipids in the second row (**c**, **d**) and for POPG lipids in the third row (**e**, **f**).

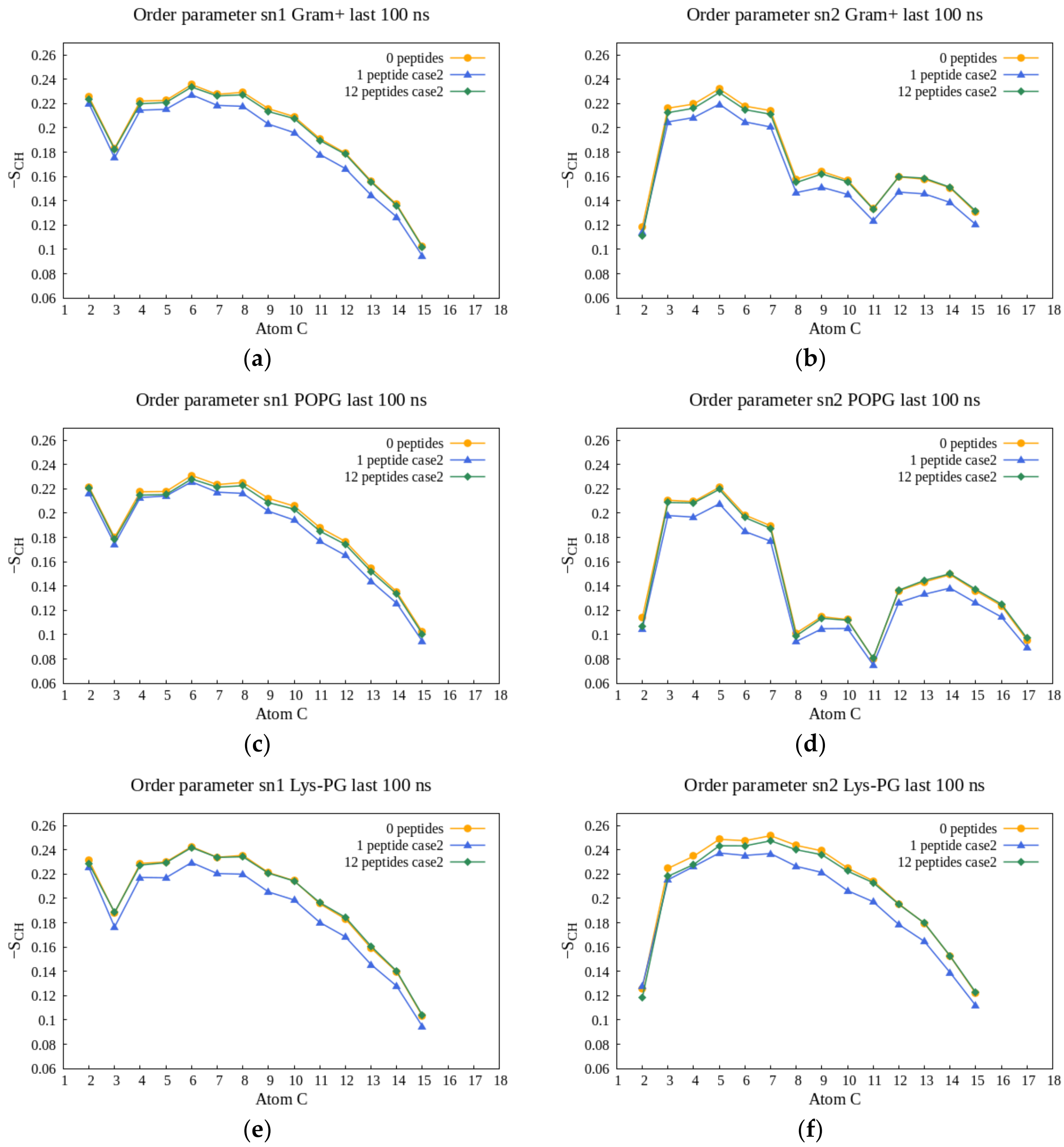

Figure S22.2. Order parameter $-S_{CH}$ for the acyl chains sn-1 (on the left) and sn-2 (on the right) as a function of the carbon atom index and calculated as averages over last 100 ns simulation time by order Gromacs utility. The legend is included in the graphs, and the results are shown for simulation cases with Gram+ membrane (AA-0, AA-1b, AA-12b), with the results for all lipids in the first row (**a**, **b**), POPG lipids in the second row (**c**, **d**) and for Lys-PG lipids in the third row (**e**, **f**).

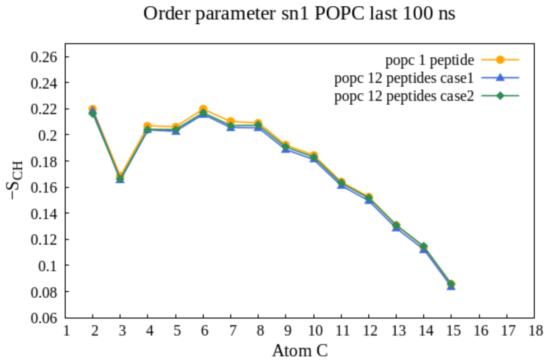

(**a**)

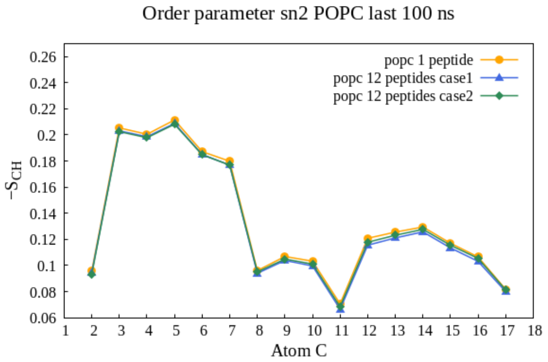

(**b**)

Figure S22.3. Order parameter $-S_{CH}$ for the acyl chains sn-1 (on the left) and sn-2 (on the right) as a function of the carbon atom index and calculated as averages over last 100 ns simulation time by order Gromacs utility. The legend is included in the graphs, and the results show POPC lipids for simulation cases with neutral membrane (AA-1, AA-12a, AA-12b).

F) Outer membrane results

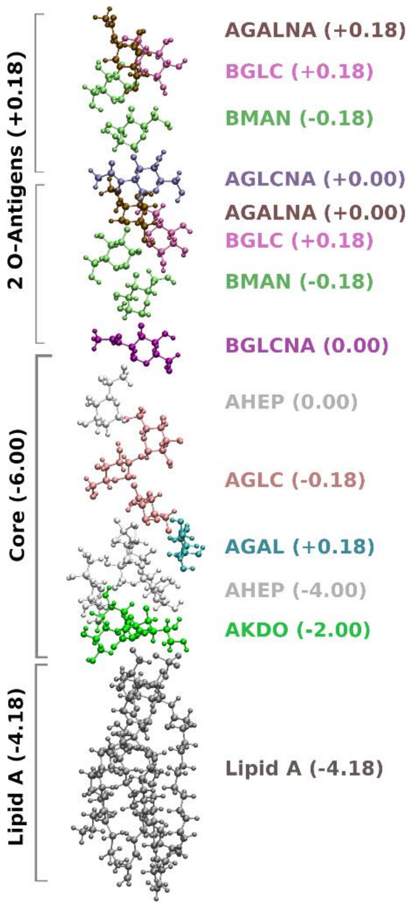

Figure S23. Structure of lipopolysaccharide (LPS) molecule with subunits charges in CHARMM force field [8]. Subunits are named according to CHARMM-GUI nomenclature [9].

Table S5. Average area per lipid, thickness and total area of lipids of outer membrane model for *E. coli* (AA-0 simulations, simulation time 500 ns) calculated by APL@Voro program.

Simulation case	Lipid	APL	Thickness	Total area
**E. coli OM 0 O-Antigens**	Lipid A	1.90	3.65	94.60
	PVPE:PVPG	0.65	3.643	87.99
	PVCL2	0.80	3.679	6.37
**E. coli OM 2 O-Antigens**	Lipid A	1.94	3.62	96.79
	PVPE:PVPG	0.67	3.624	89.79
	PVCL2	0.85	3.574	6.90

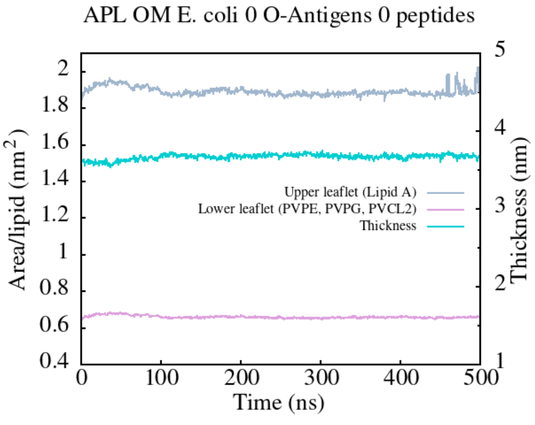

(**a**)

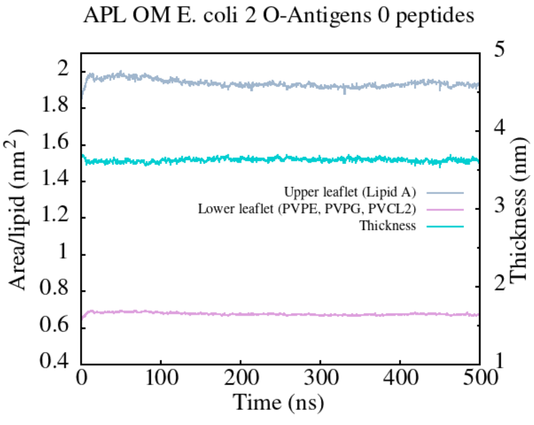

(**b**)

Figure S24. Area per lipid and membrane thickness as a function of simulation time for pure membrane systems (AA-0 simulations). The results for the rough OM (with 0 O-antigens) are shown in (**a)** and for the smooth OM (with 2 O-antigens) are shown in (**b**). Calculations are done with APL@Voro program.

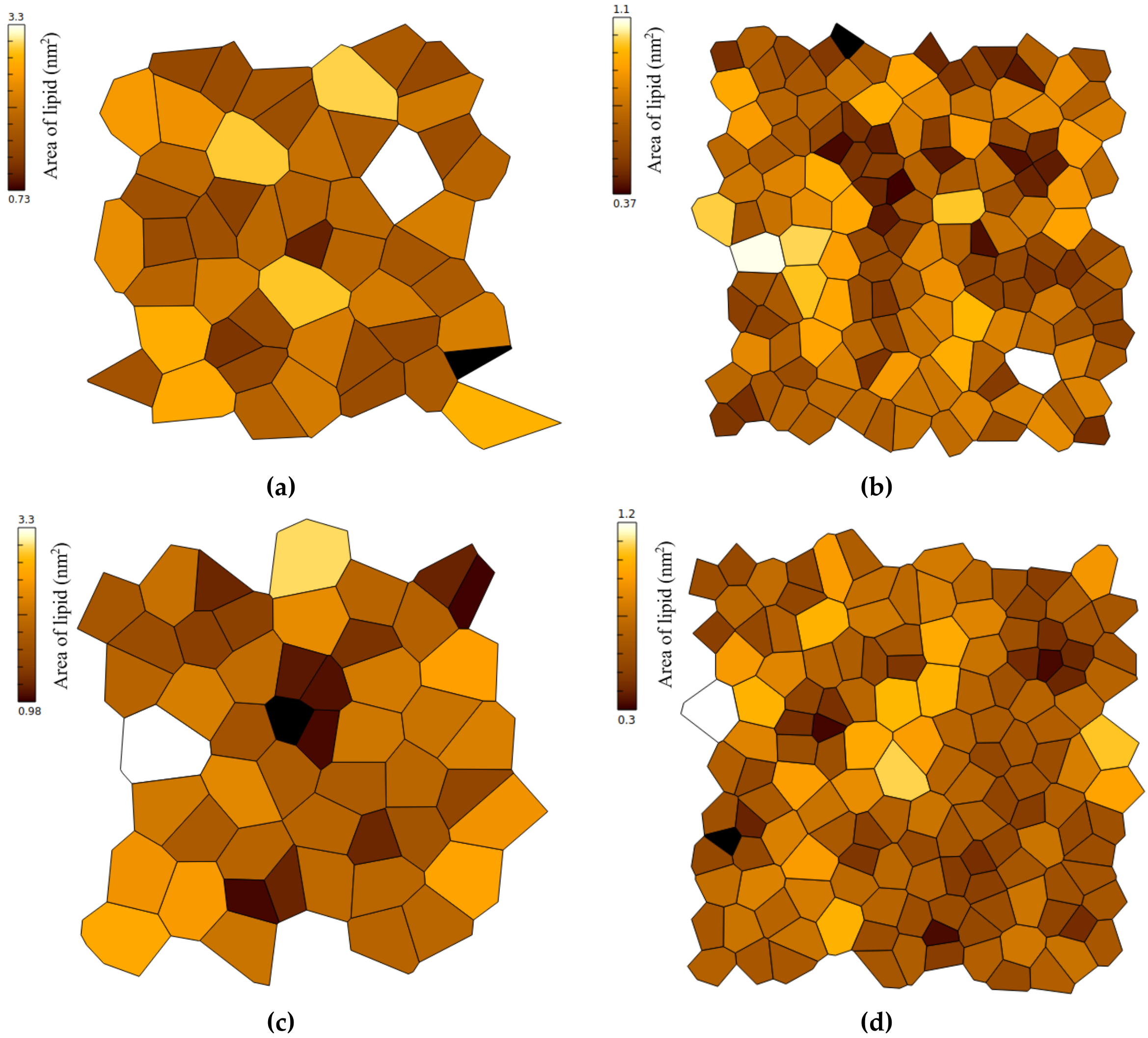

Figure S25. Area of lipids profiles calculated by APL@Voro program for the last frame of AA-0 simulation of outer membrane, the results for rough (0 O-Antigens) membrane are on the top, and for the smooth (2 O-Antigens) membrane on the bottom. On the left (**a** and **c**) is the upper leaflet (Lipid A), and on the right (**b** and **d**) lower leaflet (PVPE, PVPG and PVCL2 lipids).

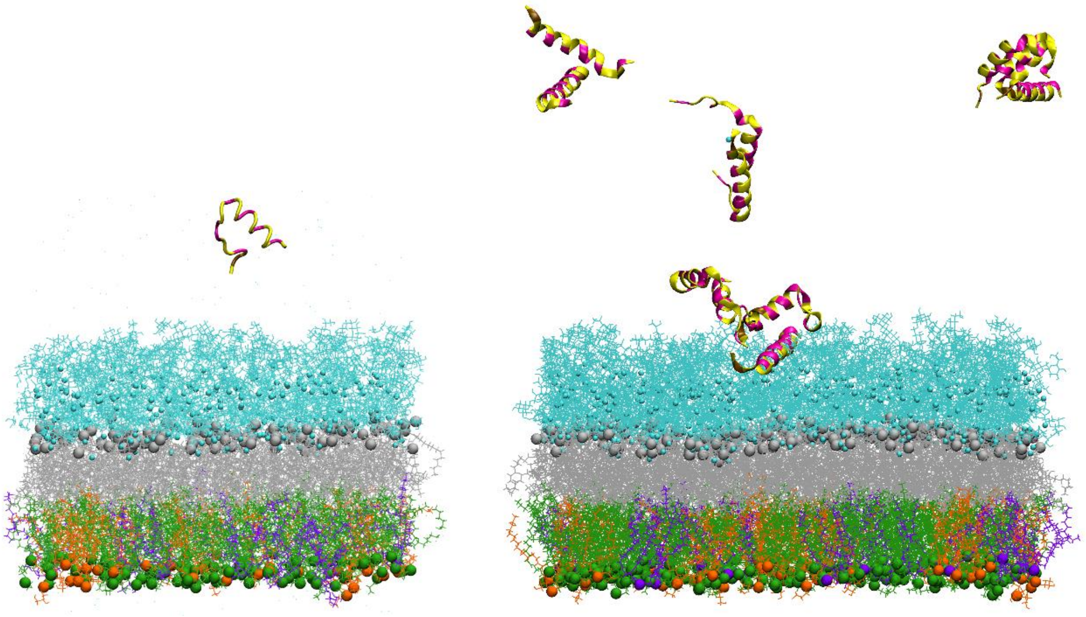

(a) **(b)**

Figure S26. Snaphshots of outer membrane of Gram-negative bacteria **without O-antigens (rough OM)** interaction with 1 peptide (**a**) and 12 peptides (**b**) at the simulation time of 100 ns. Peptides are initially placed ~2 nm above the membrane. In both cases, after ~100 ns, some of the peptides jump outside the simulation box and bind to the lower leaflet. PVPE lipids are green, PVPG lipids are orange, PVCL2 is violet, and Lipid A is gray, all represented by sticks and beads. The core region is represented by cyan lines, neutralizing Ca^{2+} ions in core region as small cyan beads. Peptides are shown as ribbons, with polar and charged residues in yellow and hydrophobic residues in magenta. Water molecules are not shown for clarity.

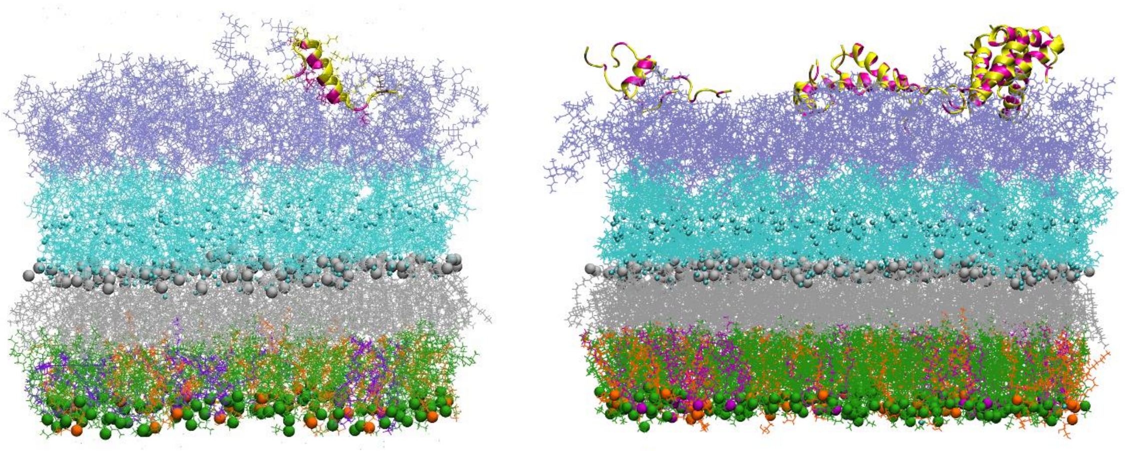

(**a**) (**b**)

Figure S27. Snapshots of outer membrane of Gram-negative bacteria **with 2 O-antigens (rough OM)** interaction with 1 peptide (**a**) and 12 peptides (**b**) at the simulation time of 500 and 400 ns respectively. Peptides are initially placed ~2 nm above the membrane. All peptides mostly stay bonded to membrane during simulation time. PVPE lipids are green, PVPG lipids are orange, PVCL2 is violet, and Lipid A is gray, all represented by sticks and beads. The core region is represented by cyan lines, neutralizing Ca^{2+} ions in core region as small cyan beads and the 2 O-antigen units are represented by purple lines. Peptides are shown as ribbons, with polar and charged residues in yellow and hydrophobic residues in magenta. Water molecules are not shown for clarity.

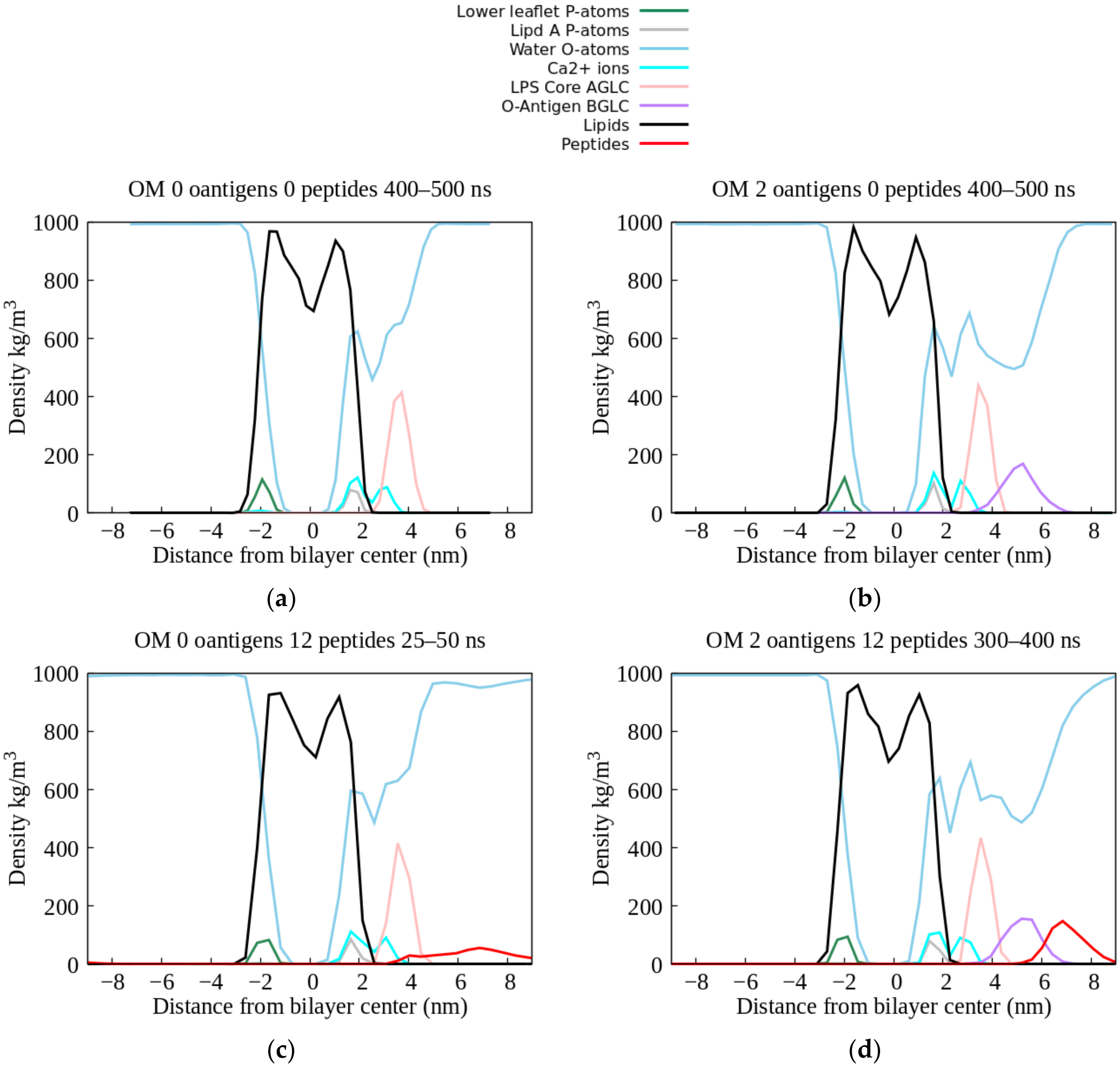

Figure S28. Density profiles of outer membrane components averaged over denoted simulation time, calculated by Gromacs density tool. The first row shows the results for pure outer membrane simulations, without O-antigens (**a**) and with 2 O-antigens (**b**). The second row displays the results of simulations of the outer membrane with 12 peptides, without O-antigens (**c**) and with 2 O-antigens (**d**). The legend is placed above the plots.

G) DSSP profiles of peptide's secondary structures

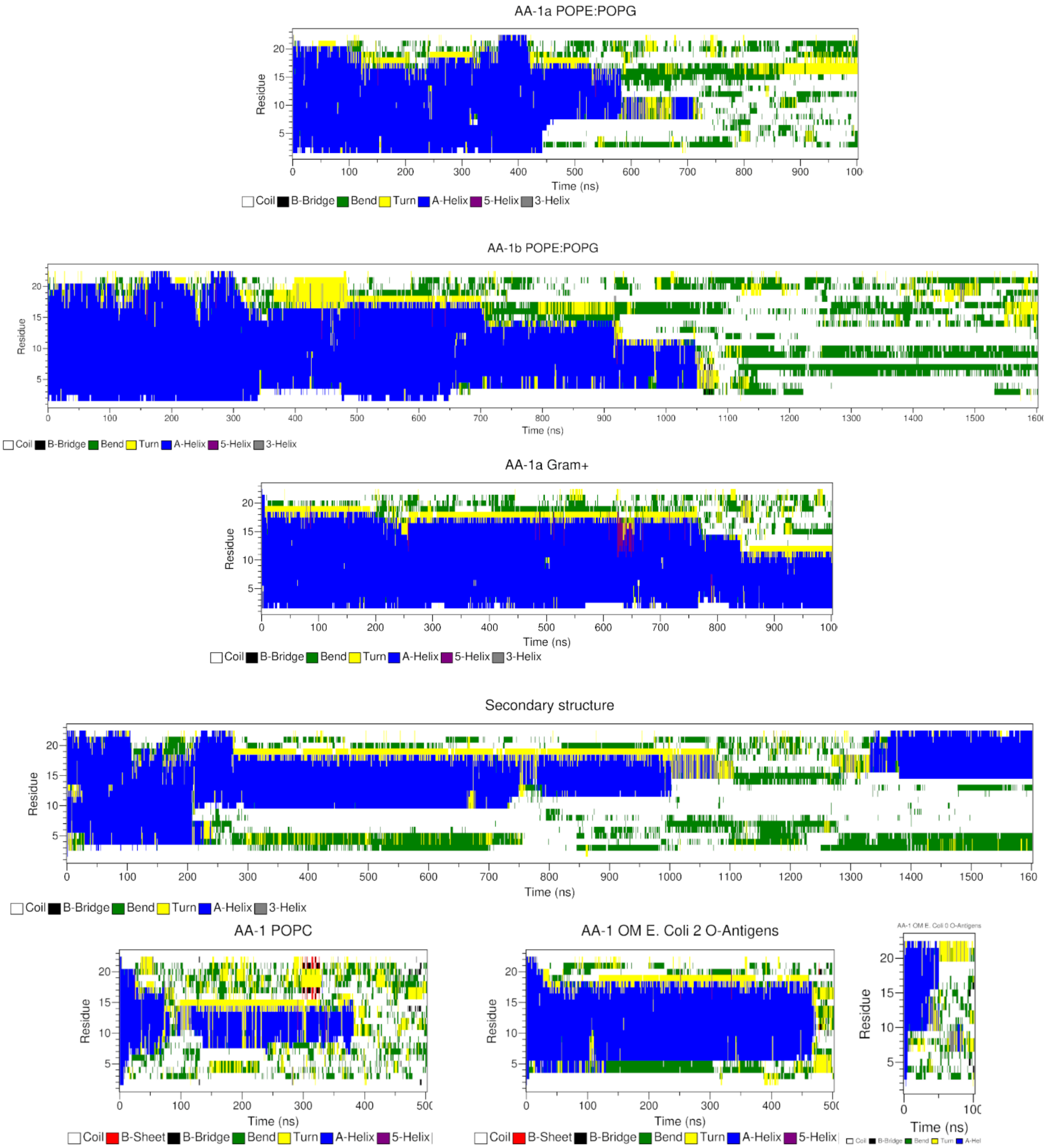

Figure S29. Secondary structure of peptides in AA-1 simulations.

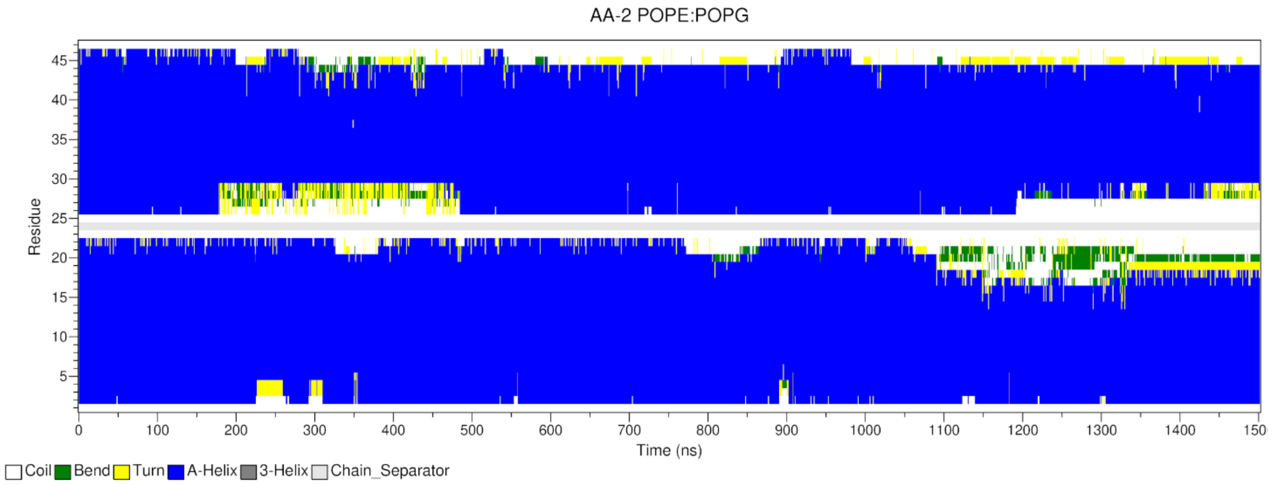

(a)

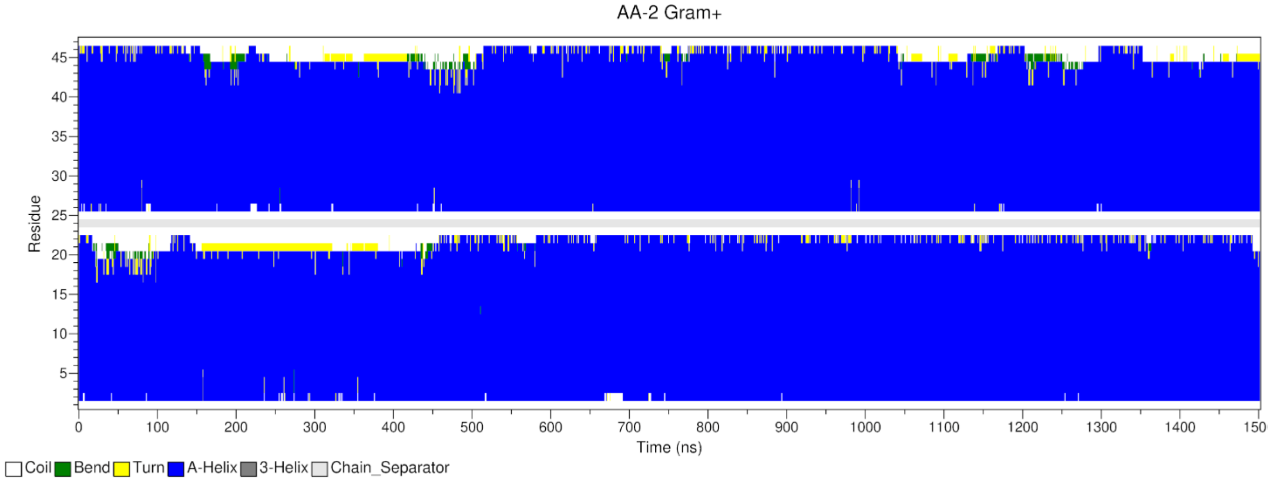

(b)

Figure S30. Secondary structure of peptides in AA-2 simulations.

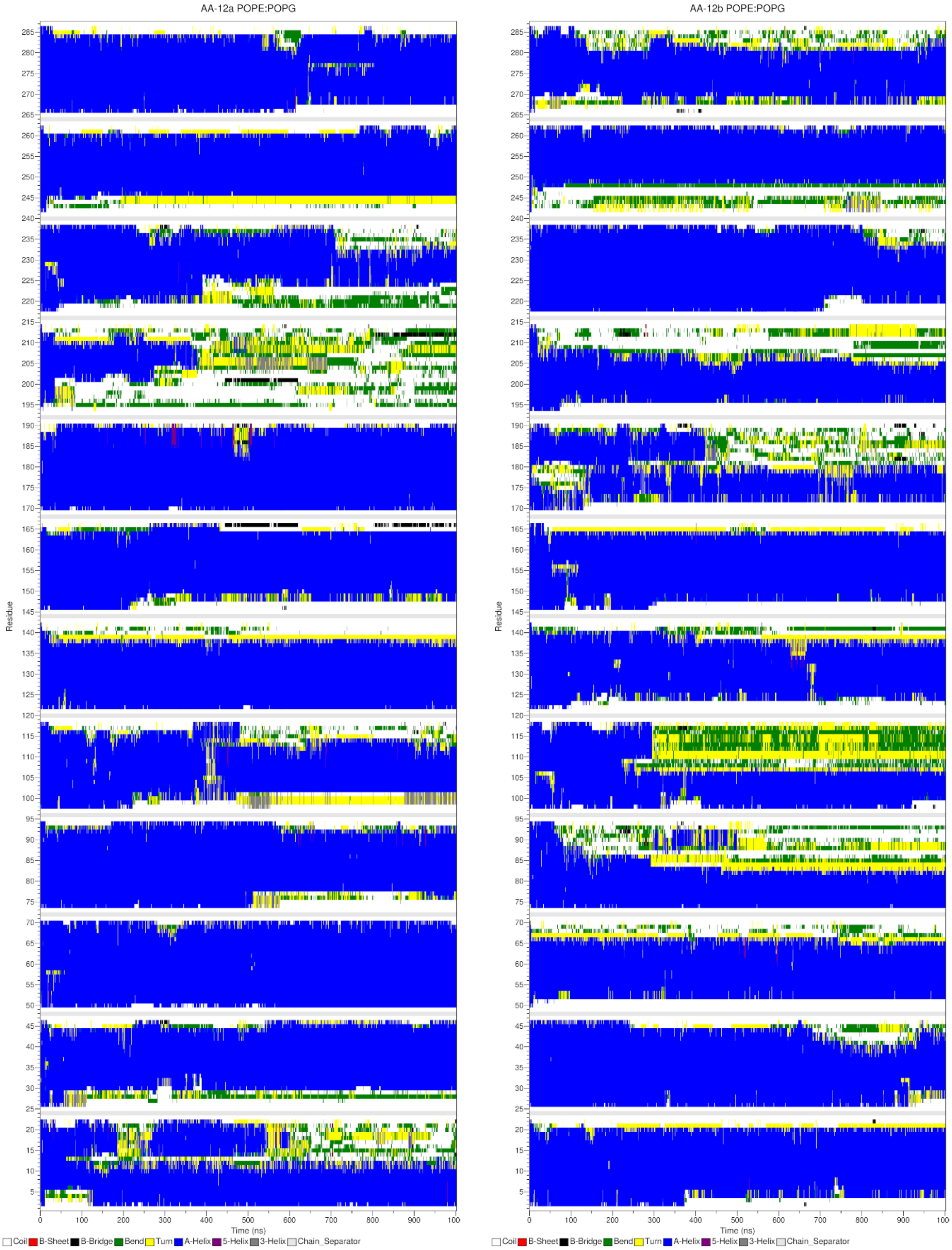

(a) **(b)**

Figure S31. Secondary structure of peptides in AA-12 POPE:POPG simulations. Case1 (**a**), case2 (**b**).

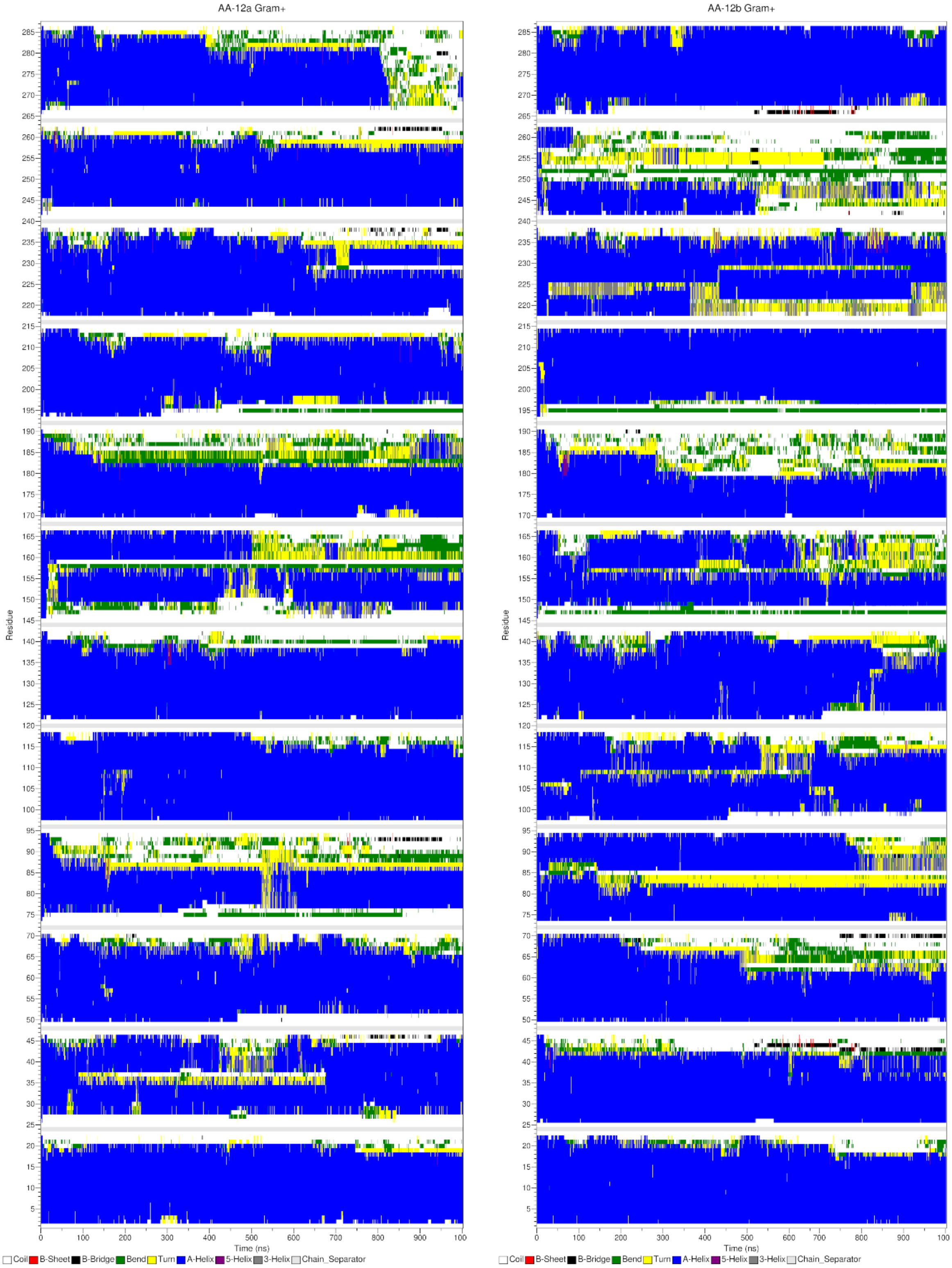

(a) **(b)**

Figure S32. Secondary structure of peptides in AA-12 PG:Lys-PG:CL simulations. Case1 (**a**), case2 (**b**).

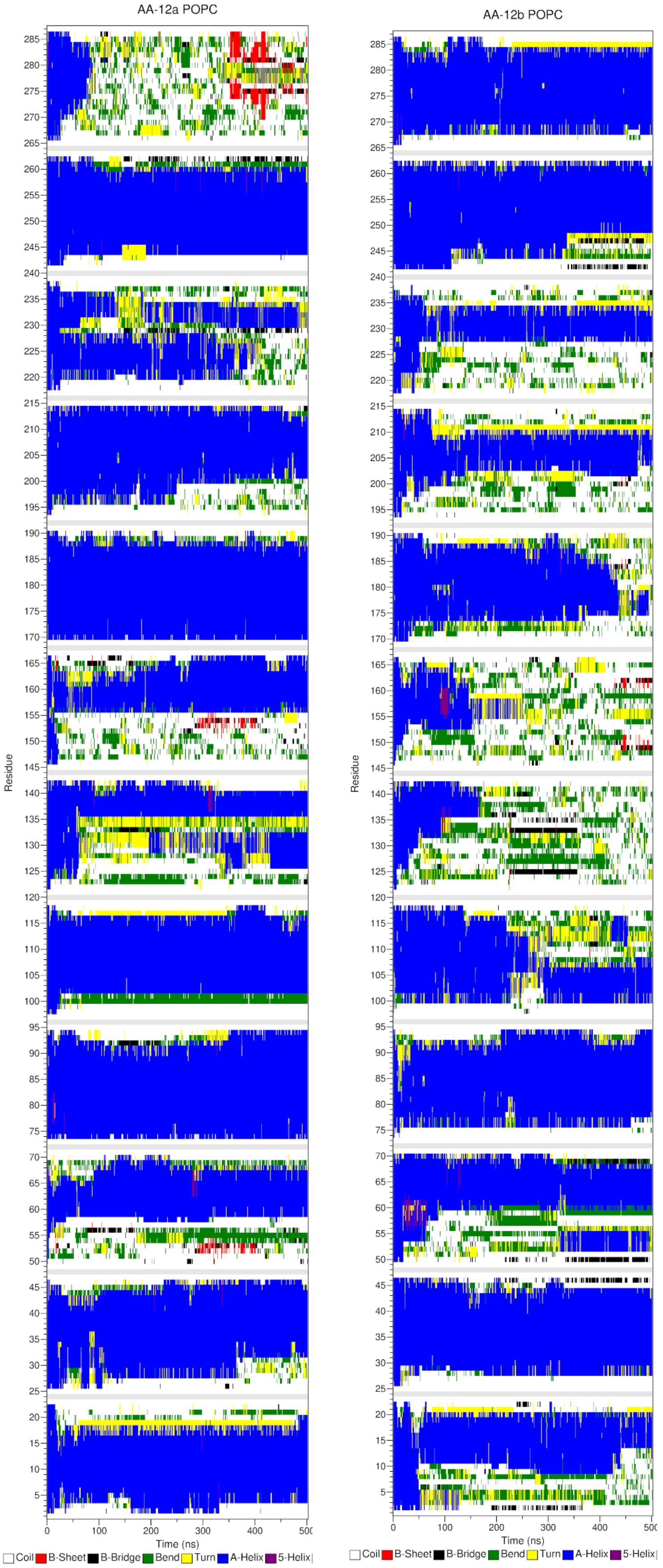

(a) **(b)**

Figure S33. Secondary structure of peptides in AA-12 POPC simulations. Case1 (**a**), case2 (**b**).

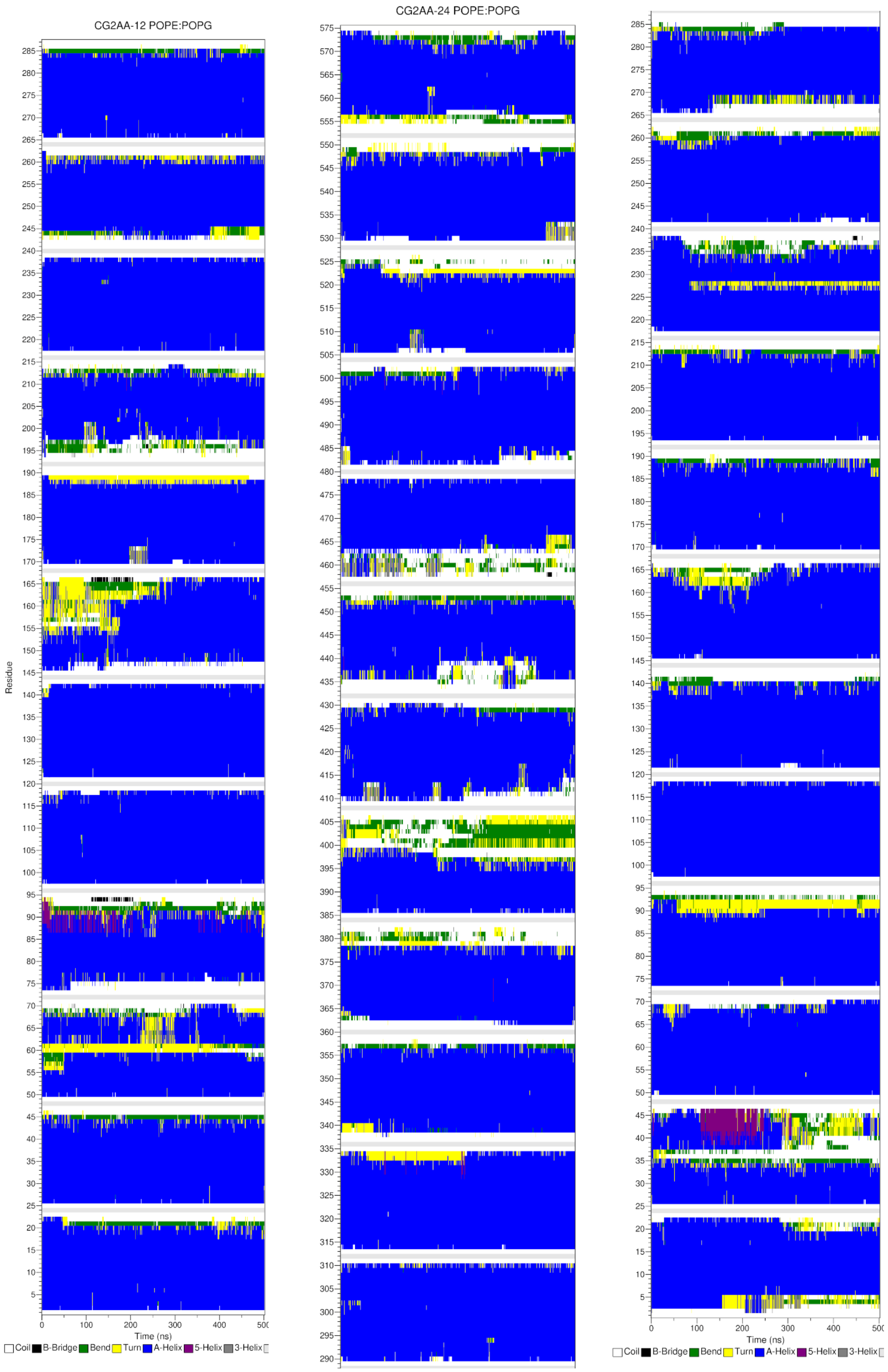

(a) **(b)**

Figure S34. Secondary structure of peptides in CG2AA-12 (**a**) and CG2AA-24 (**b**) POPE:POPG simulations.

References

1. Kucerka, N.; Van Oosten, B.; Pan, J.; Heberle, F.; Harroun, T.; Katsaras, J. Molecular Structures of Fluid Phosphatidylethanolamine Bilayers Obtained from Simulation-to-Experiment Comparisons and Experimental Scattering Density Profiles. *J. Phys. Chem. B* **2014**, *119*, 1947–1956, https://doi.org/10.1021/jp511159q.
2. Pan, J.; Heberle, F.A.; Tristram-Nagle, S.; Szymanski, M.; Koepfinger, M.; Katsaras, J.; Kučerka, N. Molecular structures of fluid phase phosphatidylglycerol bilayers as determined by small angle neutron and X-ray scattering. *Biochim. et Biophys. Acta (BBA) - Biomembr.* **2012**, *1818*, 2135–2148, https://doi.org/10.1016/j.bbamem.2012.05.007.
3. Kučerka, N.; Nieh, M.-P.; Katsaras, J. Fluid phase lipid areas and bilayer thicknesses of commonly used phosphatidylcholines as a function of temperature. *Biochim. et Biophys. Acta (BBA) - Biomembr.* **2011**, *1808*, 2761–2771, https://doi.org/10.1016/j.bbamem.2011.07.022.
4. Piggot, T.J.; Holdbrook, D.A.; Khalid, S. Electroporation of the E. coli and S. Aureus Membranes: Molecular Dynamics Simulations of Complex Bacterial Membranes. *J. Phys. Chem. B* **2011**, *115*, 13381–13388, https://doi.org/10.1021/jp207013v.
5. Murzyn, K.; Róg, T.; Pasenkiewicz-Gierula, M. Phosphatidylethanolamine-Phosphatidylglycerol Bilayer as a Model of the Inner Bacterial Membrane. *Biophys. J.* **2005**, *88*, 1091–1103, https://doi.org/10.1529/biophysj.104.048835.
6. Mortuza, S.M.; Zheng, W.; Zhang, C.; Li, Y.; Pearce, R.; Zhang, Y. Improving fragment-based ab initio protein structure assembly using low-accuracy contact-map predictions. *Nat. Commun.* **2021**, *12*, 1–12, https://doi.org/10.1038/s41467-021-25316-w.
7. Jo, S.; Lim, J.B.; Klauda, J.B.; Im, W. CHARMM-GUI Membrane Builder for Mixed Bilayers and Its Application to Yeast Membranes. *Biophys. J.* **2009**, *97*, 50–58, https://doi.org/10.1016/j.bpj.2009.04.013
8. Wu, E.L.; Cheng, X.; Jo, S.; Rui, H.; Song, K.C.; Dávila-Contreras, E.M.; Qi, Y.; Lee, J.; Monje-Galvan, V.; Venable, R.M.; et al. CHARMM-GUI Membrane Builder toward realistic biological membrane simulations. *J. Comput. Chem.* **2014**, *35*, 1997–2004, https://doi.org/10.1002/jcc.23702.
9. Jo, S.; Kim, T.; Im, W. Automated Builder and Database of Protein/Membrane Complexes for Molecular Dynamics Simulations. *PLoS ONE* **2007**, *2*, e880, https://doi.org/10.1371/journal.pone.0000880.
